# Insights into the Phosphoryl Transfer Mechanism of Human Ubiquitous Mitochondrial Creatine Kinase

**DOI:** 10.1038/srep38088

**Published:** 2016-12-02

**Authors:** Quanjie Li, Shuai Fan, Xiaoyu Li, Yuanyuan Jin, Weiqing He, Jinming Zhou, Shan Cen, ZhaoYong Yang

**Affiliations:** 1 Institute of Medicinal Biotechnology, Chinese Academy of Medical Sciences & Peking Union Medical College, Beijing 100050, China

## Abstract

Human ubiquitous mitochondrial creatine kinase (uMtCK) is responsible for the regulation of cellular energy metabolism. To investigate the phosphoryl-transfer mechanism catalyzed by human uMtCK, in this work, molecular dynamic simulations of uMtCK∙ATP-Mg^2+^∙creatine complex and quantum mechanism calculations were performed to make clear the puzzle. The theoretical studies hereof revealed that human uMtCK utilizes a two-step dissociative mechanism, in which the E227 residue of uMtCK acts as the catalytic base to accept the creatine guanidinium proton. This catalytic role of E227 was further confirmed by our assay on the phosphatase activity. Moreover, the roles of active site residues in phosphoryl transfer reaction were also identified by site directed mutagenesis. This study reveals the structural basis of biochemical activity of uMtCK and gets insights into its phosphoryl transfer mechanism.

Creatine kinase (CK, EC 2.7.3.2), belonging to the guanidino kinase superfamily, plays a critical role in cellular energy circulation in vertebrates by catalyzing the reversible phosphorylation. Through the reversible conversion between creatine and phosphocreatine, which is coupled to the equilibrium between ATP and ADP, CK helps maintain energy homeostasis in tissues, which is in need of fluctuating high energy supply^1^,^2^. Because of its essential role in cell cycle regulation, CK is considered to be a potential clinical biomarker and therapeutic target of various illnesses^3^,^4^,^5^. Most vertebrate species express three cytosolic isoenzymes: muscle-type CK (MM-CK), brain-type CK (BB-CK), and the heterodimer of these two types (MB-CK), characterized by their relative isoelectric points and tissue-specificity of expression^4^. And also two mitochondrial CK isoenzymes: ubiquitous mitochondrial CK (uMtCK) and sarcomeric mitochondrial CK (sMtCK)^3^. Among them, uMtCK is a key molecule for oxidative phosphorylation and apoptosis in the mitochondria^6^. Recent studies show that uMtCK is involved in prostate or breast cancer progression^7^,^8^.

CKs share ~60% sequence identity across all species and among the isoforms^9^. To date, in the CK family including human uMtCK^10^, 13 crystal structures have been reported and can be sorted into three groups according to the substrate binding mode: the ligand-free-form^11^,^12^,^13^,^14^, the ADP-Mg^2+^-complex^15^,^16^, and the ADP-Mg^2+^·nitrate·creatine transition-state analogue complex^17^,^18^,^19^. As the first released CK structure of human origin, human uMtCK is structurally ligand-free. Its crystal structure is formed by four “banana-shaped” dimers and crystallized in an octameric fashion. The eight independent monomers are similar and each of them has two major domains: a small N-terminal domain (residues 1–95) and a larger C-terminal domain (residues 120–379), between which is a long linker region (residues 96–119). Notably, two flexible loops (residues 61–65 and 316–326), which are supposed to participate directly in the binding of substrates, point out away from the catalytic center, leaving a relatively open binding pocket in uMtCK. The overall three-dimensional structure of human uMtCK is very similar to the other known CKs determined in the “open” unliganded mode, such as human MM-CK (PDB entry:1I0E)^20^ and BB-CK (PDB entry:3DRE)^17^, chicken sMtCK (PDB entry:1CRK)^12^ and BB-CK (PDB entry:1QH4)^13^, rabbit MM-CK (PDB entry:2CRK)^11^, and bovine BB-CK (PDB entry:1G0W)^14^.

Although the biochemical and structural investigations of human uMtCK have been reported, the actual catalytic mechanism of corresponding substrate phosphorylation has not been made clear so far. The unliganded “open” structural state of uMtCK gave no details of the substrate binding mode of the “active” enzyme complex. Besides, the negatively charged cluster of E226, E227, and D228 was shown by site-directed mutagenesis to be essential for catalytic activity^10^. But the detailed roles of these residues in the catalytic activity are not clear yet. Over the past 30 years, extensive research efforts have been made to understand the catalytic mechanism of CK^21^,^22^,^23^,^24^,^25^,^26^,^27^. Milner-White and Watts firstly proposed that CK forms a quaternary CK·ADP-Mg^2+^·nitrate·creatine complex, in which the nitrate mimics the planar γ-phosphate in the transition state^28^. This was subsequently confirmed by the transition-state analogue complex structures of human BB-CK (PDB entry:3B6R)^17^, rabbit MM-CK (PDB entry:1U6R)^19^, and torpedo california MM-CK (PDB entry:1VRP)^18^. In contrast to the unliganded open form, these complexes uncovered a number of key residues involved in ligands binding and also provided useful information about the conformational changes associated with substrates binding.

In this work, a 3D model of the uMTCK-Mg^2+^-ATP-creatine complex was constructed with respect to the transition-state analogue complex structures mentioned above. The complex was then subjected to optimization and equalization using molecular dynamic (MD) simulations. Based on the equilibrated structure, we proposed the phosphoryl transfer reaction mechanism catalyzed by uMTCK, obtained by application of the hybrid density functional theory (DFT) method B3LYP. To validate the predicted catalytic roles of active site residues in the phosphorylation reaction, we also carried out site-directed mutagenesis and catalytic activity measurements of these residues. Our study on the phosphoryl transfer reaction of human uMtCK gives new insights into its catalytic mechanism by biochemical assay coupled with theoretically MD simulation and quantum chemistry calculation.

## Results and Discussion

### A 3D Model of the uMtCK·ATP-Mg^2+^·creatine complex

#### Structural stability of the complex

**Figure 1:**
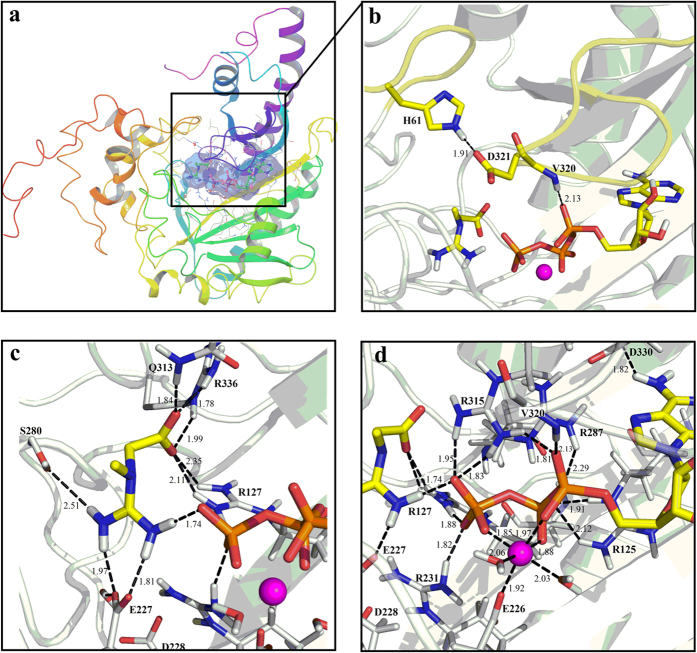
3D Model of the uMtCK•ATP-Mg^2+^•creatine complex and substrates binding. (**a**) The overall structure of the complex. The substrate binding site is shown in purple surface representation. (**b**) Two flexible loops (highlighted in yellow) remain in the “closed” reactive conformation. (**c**) A close-up view of creatine binding pocket. (**d**) A close-up view of ATP binding site. The entire protein is shown in cartoon. ATP, creatine, water, and residues interacting with them are displayed in stick and labeled. Mg^2+^ is shown as magenta sphere. Black dashed lines represent hydrogen bonds or salt bridges. Distances of H-bonds or salt bridges are given in angstroms.

Because the crystal structure of uMtCK represented the unliganded “open” state, we predicted a 3D model of uMtCK in complex with the co-factor ATP-Mg^2+^ and creatine. The structure was then solvated using TIP3P water molecules followed by energy minimization and 10 ns MD simulations to remove bad contacts. The equilibrated structure is depicted in Fig. 1a. The backbone root mean square deviation values of the simulated complex with respect to the initial structure are plotted in Supplementary Fig. S1. The corresponding values converge around 2.5 Å after 2 ns simulation and shows no more significant fluctuation afterwards, revealing that the structure remained stable during the MD simulations. The most striking difference between the uMtCK•ATP-Mg^2+^•creatine complex and the initial unliganded crystal structure is the movement of two flexible loops (Fig. 1b). Superposition of these two structures shows that both loops move into the catalytic center and form a “closed” conformation upon substrates binding (see Supplementary Fig. S2).

#### Substrate binding sites in enzymes

The active site for phosphorylation is located in the deep cleft covered by the two loops (Fig. 1b). Under the restriction of uMtCK, ATP-Mg^2+^ and creatine form a highly advantageous conformation for the biochemical reaction. Creatine has its own binding pocket, separating from that of ATP (Fig. 1c). The carboxylate group of E227 forms two H-bonds with the guanidinium moiety of creatine, which turns creatine into the catalytically favorable position. The S280 residue also interacts with creatine and helps to keep the substrate anchored and positioned for nucleophilic attack at ATP-Mg^2+^. The Q313, R336, and R127 residues form H-bonds with the carboxylate group of creatine. Most importantly, one of the oxygen atoms of P_γ_O_3_ group interacts with the guanidinium moiety of creatine through a strong H-bond (1.74 Å). This near attack position is favorable for the nucleophilic substitution reaction.

The ATP binding pocket consists of five highly conserved arginine residues (R125, R127, R231, R287, and R315), D330, V320, and Mg^2+^ ion. As seen in the equilibrated structure (Fig. 1d), Mg^2+^ ion is hexa-coordinated with α-, β-, and γ-phosphate groups of ATP, the carboxyl group of E226, and also two water molecules. As a metal ion cofactor required for the reaction, Mg^2+^ plays a role in neutralizing the negative charges of ATP and stabilizing the phosphoryl transfer reaction^29^. The arginine residues interact directly with the phosphate oxygen of ATP (R125 with O_β_, R127 with O_γ_ and the oxygen of creatine, R231 with O_γ_, R287 with the oxygen that bridges α- and β-phosphate group, R315 with O_α_ and O_γ_) and help to stabilize the negatively charged phosphate groups. Two additional interactions with ATP involve side chain of D330 and the main chain of V320. These tight H-bond interactions ensure near-attack position of creatine and ATP-Mg^2+^, which will facilitate the subsequent nucleophilic substitution.

### The phosphoryl transfer reaction mechanism catalyzed by uMtCK

**Figure 2:**
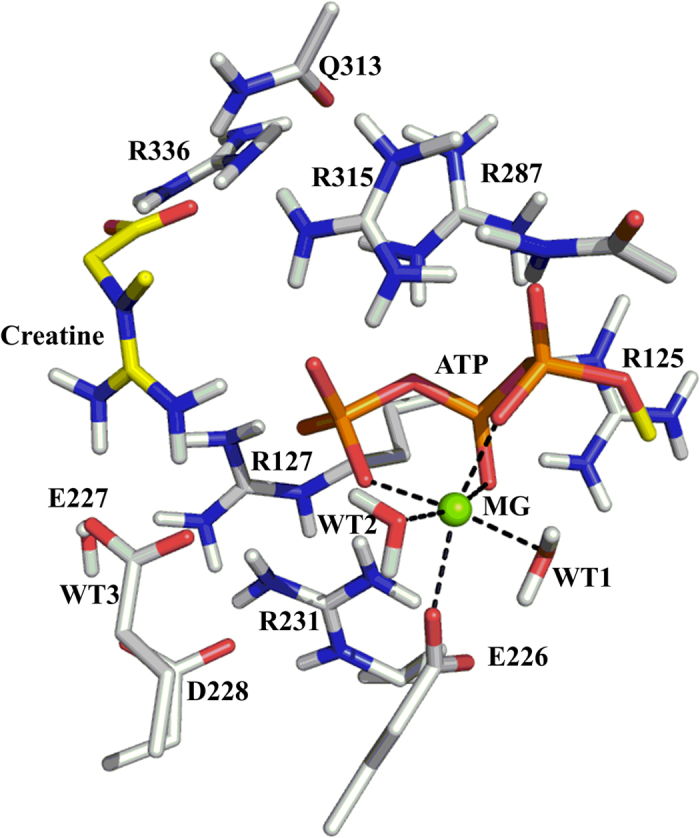
Optimized structure of the active site model of uMtCK in complex with substrate creatine and ATP-Mg^2+^. The model consists of the methyl-triphosphate arm of ATP, creatine, the Mg^2+^ ion and its first-shell coordination, including E226 and two water molecules. Nine important residues: R125, R127, R231, E227, D228, R287, Q313, R315, and R336 are also included. The substrate creatine and ATP are colored by atom type with carbon atoms in yellow. While the residues are colored in grey. For clarity, only polar hydrogen atoms were shown.

**Figure 3:**
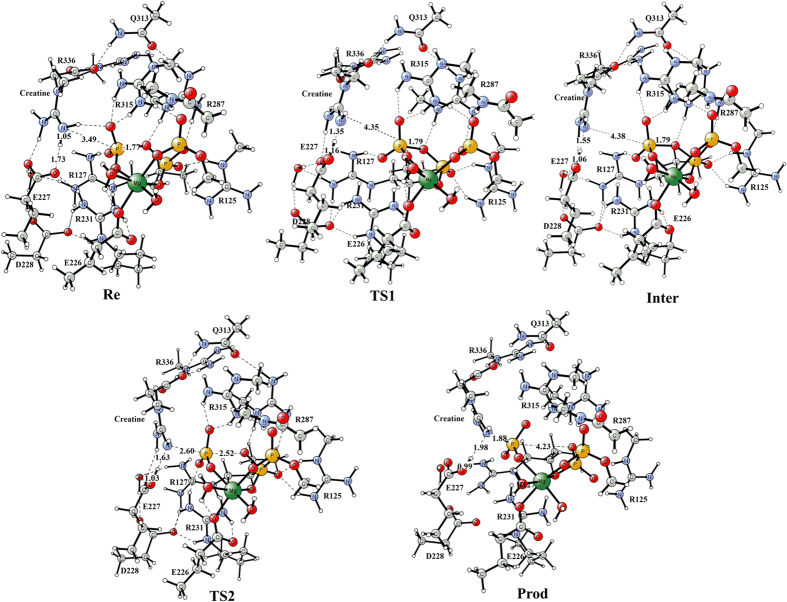
Optimized geometries of the reactant, intermediate, transition states, and product for the E227-assisted phosphoryl transfer reaction. A stepwise dissociative pathway was located on potential energy surface: (1) E227 acts as the catalytic base to receive the guanidinium proton transferred from creatine. (2) The γ-phosphate group is transferred to creatine, releasing ADP and phosphocreatine. Distances are given in angstroms.

**Figure 4:**
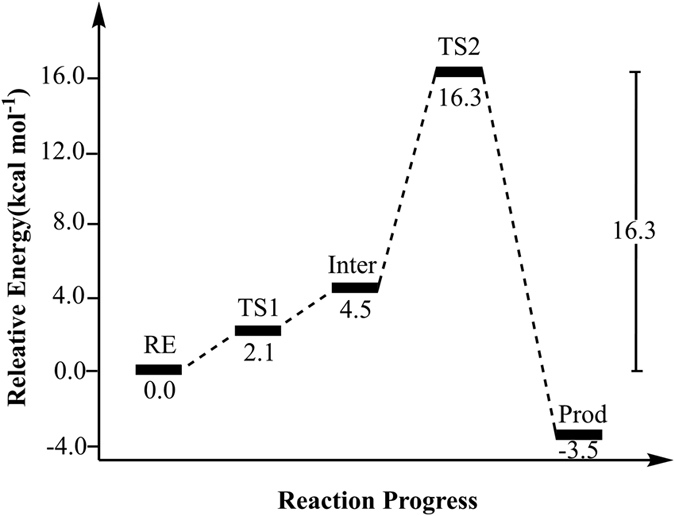
Calculated potential energy profile for the phosphoryl transfer reaction mechanism catalyzed by uMTCK. The relative free energies (unit: kcal mol^−1^) are shown for the reactant (Re), intermediate (Inter), transition states (TS1, TS2), and product (prod).

A quantum chemical model of active site was constructed based on the equilibrated structure of uMtCK•ATP-Mg^2+^•creatine complex. The optimized structure of this model was displayed in Fig. 2. Starting from it, we did a relaxed potential energy surface scan to search for reasonable transition state structures. A two-step dissociative pathway was located on potential energy surface. In the first step, creatine delivered a guanidinium proton to the carboxylate oxygen atom of E227. In the second step, the γ-phosphate group of ATP transferred to creatine in a reversible reaction that forms ADP and phosphocreatine. The second step is the rate-determining step with the calculated energy barrier of 16.3 kcal mol^−1^, which agrees well with our experimentally determined value of uMtCK (Table 1, 16.7 kcal mol^−1^). This value is also closer to the upper range of activation energies previously reported for CK isoenzymes MM-CK, MB-CK, and BB-CK (11.7~15.0 kcal mol^−1^)^30^. Geometries and the potential energy profiles calculated for this mechanism are shown in Figs 3 and 4, respectively. Cartesian coordinates for all these structures can be found as Supplementary Data.

In the optimized pre-reactive complex (Fig. 3, Re), the nucleophilic nitrogen atom of creatine guanidine group is 3.49 Å away from P_γ_-ATP. In addition, the hydrogen of the creatine guanidine group forms a short H-bond (1.73 Å) with the carboxylate oxygen atom of E227. Clearly, this near-attack conformation of substrates observed in the reactant is favorable for the nucleophilic attack at phosphorus with the guanidinium proton transfers to E227. Besides, the reaction model is also stabilized by the H-bond network involving R127, E227, D228, R231, and the γ-phosphate group.

These reactive events first take place via transition state 1 (Fig. 3, TS1). The transition vector of TS1 (ν = −531 i cm^−1^) is dominated by the proton transfer between the guanidinium group and the carboxylate oxygen atom of E227. The distance of Creatine-NH∙∙∙H outstretched from 1.06 Å in Re to 1.35 Å in TS1. The Creatine-N∙∙∙P_γ_ distance (4.35 Å) became longer than that of Re (3.49 Å). Other non-covalent interactions in TS1 remained largely unchanged. The calculated energy of TS1 is 2.1 kcal mol^−1^ higher than that of Re. After passing through TS1, the positively charged guanidinium group of creatine donated its proton to the carboxylate oxygen atom of E227 and produced the intermediate structure (Fig. 3, Inter). This is favorable for the next nucleophilic displacement. The resulted Creatine-NH and E227-COOH group interacted strongly via a short H-bond (1.55 Å). Inter lies 2.4 kcal mol^−1^ higher in energy than TS1 and is able to reversibly rearrange back to the reactive complex essentially without a barrier. That TS’s relative energy is lower than Inter’s is a common artifact of the use of zero-point vibrational energy (ZPVE) correction and/or empirical corrections on flat potential energy surface^31^,^32^,^33^. Interestingly, in our specific system, the stabilization effect on TS1 from ZPVE correction happens to be more significant than that of Inter. The energy of TS1 is 0.4 kcal mol^−1^ higher than that of Inter when the energy corrections are not taken into account.

The second step is the rate-determining step with the calculated energy barrier of 16.3 kcal mol^−1^. As shown in Fig. 3, the distance of Creatine-N_γ_–P_γ_ decreased from 4.38 Å in the Inter to 2.60 Å in TS2, while the P_γ_ –O_3β_ was outstretched from 1.77 Å to 2.52 Å. The bond lengths indicate that uMtCK may follow a dissociative catalytic mechanism. This dissociative characteristic of TS2 can be also calculated using Pauling’s formula: D (n) = D (1)−0.6logn^34^. Here, D (1) = 1.73 Å for the P-O bond, and D (n) is defined as the average of the two P–O distance (2.56 Å) at the translation state. The fractional bond number (n) is thus 0.04, which gives a dissociative character of 96%. Frequency calculation confirmed that the transition vector at TS2 (ν = −157 i cm^−1^) is dominated by the nucleophilic displacement.

In the product complex obtained from the phosphoryl-transfer reaction (Fig. 3, Pro), the γ-phosphate group transferred to the guanidinium moiety of creatine (Creatine-N_γ_–P_γ_ = 1.88 Å). The optimized distance of P_γ_ –O_3β_ (4.23 Å) is longer than the corresponding van der Walls distance (3.3 Å), indicating that the P_γ_–O_3β_ is completely broken. In this structure, the protonated carboxylate group of E227 still interacted closely with the Creatine-N_γ_ through a short H-bond (1.98 Å). Energetically, Pro is more stable than Re by 3.5 kcal mol^−1^.

Similar to the other kinases, our results revealed that Mg^2+^ ion plays key role in the catalytic reaction of CK. Mg^2+^ contributes to neutralizing the negatively charged triphosphate tail of ATP and stabilizing the transition state. As shown in both Figs 1c and 3, Mg^2+^ ion located at the center of the reaction system, 6-fold coordinated by E226, α-, β-, γ-phosphate groups and two water molecules throughout the reaction. This 6-fold coordination of Mg^2+^ is essential for the phosphorylation reaction^35^. Our observation is in agreement with Hirano’s previous studies^36^. They investigated the catalytic mechanism of cAMP-dependent protein kinase with the assistance of a single Mg^2+^ ion and found that only when Mg^2+^ is 6-fold coordinated, could the release of the phosphorylated substrate could occur.

### Catalytic roles of the critical active-site residues

The E227 residue forms bidentate interactions with creatine in the uMtCK·ATP-Mg^2+^·creatine complex (Fig. 2c), analogous to E225 in arginine kinase^37^, and E232 in the transition-state analogue complex structures of torpedo california MM-CK and human BB-CK. In addition to stabilizing the positively charged cluster model, E227 acts as a general base catalyst to abstract the proton from the nucleophilic nitrogen of the creatine guanidino group during the phosphoryl transfer reaction. This was confirmed by both DFT calculations and enzymatic activity assay for uMtCK. As seen in Fig. 3, the side chain of E227 interacts with the nucleophilic nitrogen through a short H-bond (1.73 Å) in the reaction state. Then the proton transfered to one of the carboxylate oxygen atoms of E227 through the TS1 structure. After that, the protonated side chain of E227 still forms H-bond interaction with creatine in the following phosphoryl transfer process. Remarkably, the proton transfer occurs prior to the γ-phosphate release, thus facilitating the nucleophilic attack by the substrate.

**Figure 5:**
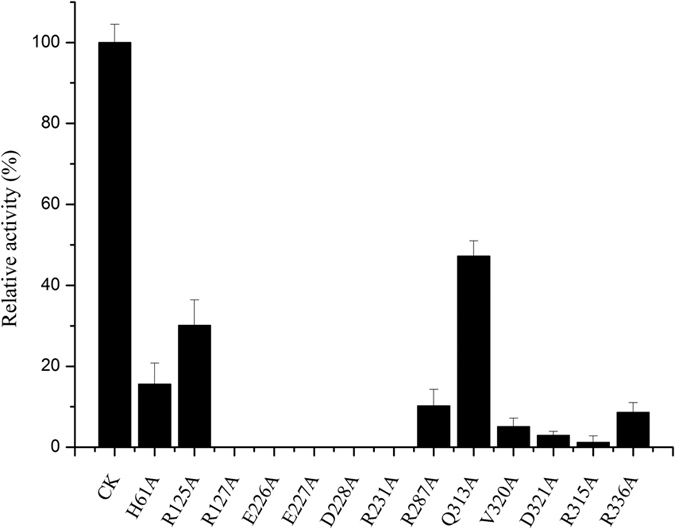
Results of uMtCK and mutant activity assay. The enzymatic activity of the purified uMtCK and mutants were measured at 30 °C with the substrate. The activity of uMtCK was determined as described by the creatine kinase assay kit (Jiancheng Nanjing Institute of Bio-Engineering, China). The original activity of uMtCK was defined as 100%. (p < 0.05).

In our site-directed mutagenesis studies (Fig. 5 and Table 2), substitution of the E227 by alanine completely abolished kinase activity, and even the conservative mutation E227D results in a 260-fold loss of activity. To further study the influence of E227 on the kinase activity, we also performed 10 ns MD simulation of E227D mutant in complex with the co-factor ATP-Mg^2+^ and creatine. The most striking structural difference between this mutant and the wild-type uMtCK is that D227 located far away from creatine and did not form H-bond with the nucleophilic nitrogen (Fig. S4). In particular, the average distances between Nγ atoms of creatine and the carboxylate oxygen atoms of D227 range from 3.9 Å to 4.5 Å, which is unfavorable for the reaction. As mentioned above, the strong H-bonds formed between creatine and the negatively charged carboxylate group of E227 stabilize the positively charged guanidinium moiety, which will facilitate the subsequent nucleophilic attack at phosphorus. Based on the equilibrated structure of E227D mutant, we also built a cluster model of the active center for DFT investigation. Despite significant efforts, we were unable to locate reasonable transition state structures for the reaction catalyzed by E227D mutant. This indicates that E227 is an indispensable residue during the phosphorylation reaction.

In addition to E227, the other two residues (E226, D228) in the negatively charged cluster are also essential for the catalytic activity. As shown in Fig. 5, the E226A and D228A mutant derivatives caused the complete loss of kinase activity. In DFT studies, E226 coordinated with the Mg^2+^ ion throughout the reaction. E226A would abolish the 6-fold coordination of Mg^2+^ ion and make it more difficult for the release of the phosphorylated substrate. Although D228 did not interact with the substrates directly, by being part of the H-bond network involving R127, E227, D228, R231, and P_γ_O_3_ group, the negatively charged D228 appeared to provide electrostatic stabilization to the condensed positive charges of the reaction model.

There are five highly conserved arginine residues (R125, R127, R231, R287, and R315) involved in ATP binding site. They interact directly with the nucleotide phosphate and mainly help stabilize the negatively charged phosphate groups. As seen in Fig. 3, R125, R287, and R315 form H-bonds with three phosphate groups throughout the reaction. Substituting these three arginine residues with alanine caused a marked decrease in enzyme activity (Fig. 5). Compared with the WT, 30.1%, 10%, and 2% of the CK activity were detected for R125A, R287A, and R315A, respectively. Notably, R127 and R231 were involved in the H-bond network mentioned above. They interacted with creatine and the γ-phosphate group directly or indirectly via one or more bridging molecules. The strong hydrogen bond interactions enhance the stability of the cluster model, which is favorable to the reaction. R127A and R231A resulted in the total loss of uMtCK kinase activity.

V320 on the loop (residues 316–326) forms a H-bond (2.13 Å) with the α-phosphates of ATP (Fig. 1b). H61, part of the conserved PGHP motif of CK found in loop (residues 61–65), has been reported to be important for the catalytic reaction^10^. Our equilibrated structure also shows that H61 interacts with the carboxyl group of D321 on the loop 316–326, effectively “latching” the two loops into the “closed” conformation. Complete “unlatching” the loops with the mutation produces loss in activity. Compared with the WT, 4.0% and 26% of the CK activity were detected for D321A and H61A respectively (Fig. 5). The same conformational changes are also observed in the transition-state analogue complex structures of human BB-CK and Torpedo California MM-CK (PDB entry: 1VRP). In these structures, H66 (loop 60–70) interacts with D326 (loop 323–332), consequently closing the two loops upon substrates binding.

## Conclusion

We predicted a 3D structure of the uMtCK•ATP-Mg^2+^•creatine complex using MD simulation method. The generated model provide critical features required for uMtCK activation, including the “closed” conformation of two flexible loops and also the reasonable near-attack position of the substrates. This structure was used as a good starting point to investigate the phosphoryl transfer reaction catalyzed by uMtCK. As a result of DFT calculations, a dissociative kinase reaction mechanism was characterized for uMtCK. The calculated energy barrier agrees well with our experimentally determined activation energy. Prior to the phosphoryl transfer step, the highly conserved residue E227 acts as the catalytic base to accept the guanidinium proton transferred from creatine. This indispensable role of E227 was further confirmed by our assay on the phosphatase activity. Considering the critical role of uMtCK in human health and disease, the catalytic mechanism studies of uMtCK should be useful for medicinal investigations.

## Methods

### Construction of a ternary complex

In order to build a 3D structure of the uMtCK•ATP-Mg^2+^•creatine complex, we extracted chain A from the crystal structure of human uMtCK (PDB ID 1QK1) and deleted the phosphate group and all the water molecules. After structural alignment with the transition-state analog complex, ATP, Mg^2+^ and creatine were manually placed into the catalytic site according to the orientation of ligands in the transition-state analogue complexes (PDB ID 1U6R and 1VRP).

### MD simulations

MD simulations of the uMtCK•ATP-Mg^2+^•creatine complex were performed using Amber11 software package^38^. The amber ff99SB force field^39^ was applied for the protein. The electrostatic potentials of ATP and creatine were determined by restrained electrostatic potential charge fitting from ref. 40 QM calculation at B3LYP/6–31 G* level. The remaining force field parameters for ligands were assigned generalized Amber force field (GAFF)^41^. TIP3P water molecules^42^ were utilized to solvate the complex, extending at least 10 Å from the protein. The box volume is 81.06 × 87.92 × 104.37 Å^3^. Two Na^+^ ions were added for charge neutralization. To remove the bad contacts, the system was subjected to energy minimization. Firstly, the water molecules and ions were refined through 2,500 steps of steepest descent followed by 2,500 steps of conjugate gradient, keeping the protein and ligands fixed. Secondly, the whole system was relaxed by 10,000 cycles of minimization procedure with 5,000 cycles of steepest descent and 5,000 cycles of conjugate gradient minimization. After that, the system was heated from 0 to 310 K by 500 ps position restraint simulation. A 2 ns MD simulations without any restraints were sequentially performed to equilibrate the complex. Finally, a length of 10 ns trajectory was computed at 310 K under constant pressure. During the simulation, the cutoff distance for van der Waals interactions was set to 10.0 Å, along with a time step of 2.0 fs. Long-range electrostatic interactions are treated with the Particle Mesh Ewald (PME) method^43^,^44^. The SHAKE algorithm^45^ was applied to constrain all bonds involving hydrogen atoms. All of the MD results were analyzed using the ptraj module of the Amber 11 software package.

### Generation of a cluster model for quantum mechanism calculation

After 10 ns simulation, a quantum chemical model of active site was constructed from the catalytic center to investigate the phosphoryl transfer mechanism of uMtCK. The model consists of the methyl-triphosphate arm of ATP, creatine, the Mg^2+^ ion and its first-shell coordination, including E226 and two water molecules. Eight important residues, E227, R231, R125, R127, R287, Q313, R315 and R336 were also included in the model. Mg^2+^ is coordinated by the conserved residue E226, α-, β-, γ-phosphate groups and two water molecules. The amino acids were truncated such that arginines, aspartates, glutamates, and glutamine were represented by methyl-guanidine, propionates, butyrates, and aceamide respectively. Notably, R127 and R231 were represented by butyl-guanidine. These residues interacted with both creatine and the γ-phosphate group directly or indirectly via one or more bridging molecules, so we kept the full side chains of them in this model. The truncation atoms were saturated by hydrogen atoms and kept fixed to their positions from the equilibrated uMtCK•ATP-Mg^2+^•creatine complex structure. The total size of the model is 198 atoms, and the total charge is +1.

### DFT calculations

Geometry optimizations as well as transition state search were carried out by using the hybrid density functional B3LYP with 6–31 G (d) basis sets^46^,^47^,^48^. Based on the located stationary point geometries, high accurate energies were further evaluated by the B3LYP with larger and nearly saturated basis sets (6–311+G (d)^49^ for Mg^2+^ and cc-pvtz (-f)^50^ for the rest. Because the polarization effects of the protein environment are not explicitly included in the quantum chemical model, we estimated it by using the CPCM continuum solvation model. The dielectric constant and the a probe radius were set to 4.0 and1.4 Å, respectively. Solvent Accessible Surface was also specified during the calculation. Zero point energy (ZPE) effect correction was performed at the same level as the geometry optimizations. As discussed above, the truncation atoms were fixed during geometry optimization. This results in a few small imaginary frequencies, on the order of 5–35 cm^−1^, existed during the frequency calculations. These imaginary frequencies do not significantly contribute to the zero-point energy correction and can be tolerated. The van der Waals effect was calculated by B3LYP-D using Grimmer’s D2 method^51^ implemented in XYZ-Viewer. All quantum mechanism calculations were performed using the Gaussian09 program^52^.

### Chemicals, Bacterial strains and plasmids

Creatine was purchased from Sigma-Aldrich. All other chemicals of the highest analytical grade were locally obtained. *Escherichia coli* BL21 (DE3) (Novagen) and pET-28a (+) (Novagen) were used for creatine kinase expression.

### Cloning and site-directed mutagenesis of uMtCK

Synthetic human ubiquitous mitochondrial CK gene (GenBank accession J04469) was custom synthesized by Genscript with codon-optimization for *E. coli* host. Using the polymerase chain reaction (PCR), the gene was amplified with the primer pairs, uMtCK-F (CGGGATCCGCAGCATCAGAACGTCGCCG) and uMtCK-R (CCCAAGCTTTCAATGTTTCGTATGAATCACCGG) which were incorporated into BamH I and Hind III restriction sites (underlined), respectively. The PCR product was purified, digested with the corresponding enzymes and cloned in the pET-28a (+) to construct the recombined pET-28CK plasmid. Site-directed mutagenesis of the amino acids in the human ubiquitous mitochondrial CK gene was achieved by overlapping extension PCR^53^ to make recombined pET-28mutant plasmid. After the verification of the sequences, these recombined plasmids were transformed respectively into the competent BL21 (DE3) for heterologous expression. All oligonucleotide primers are listed in Supplementary Table S1.

### Expression and purification of the uMtCK and mutant

The described *E. coli* strains were cultivated in LB-medium containing 50 μg ml^−1^ kanamycin at 37 °C and 200 rpm overnight, and then, 12 mL of the culture was reinoculated into 3 L shake flasks using 600 mL LB-media. The recombinant uMtCK and mutant expression was achieved by induction with 0.1 mM IPTG, when the culture reached an OD_600_ of 0.8–1.0, and the cells were cultivated at 20 °C with shaking at 200 rpm for 12 h. The cells were harvested by centrifugation at 5000 × g for 10 min at 4 °C and resuspended in 50 mL lysis buffer (20 mM phosphate buffer, 150 mM NaCl, 20 mM imidazole, 1 mM DTT, pH 7.4). The cells were disrupted with a high pressure homogenizer and the cell debris were removed by centrifugation at 16000 × g for 30 min at 4 °C. The soluble fraction was passed through a 0.45 μm syringe filter unit, and then applied to a Ni-NTA agarose gel column (Qiagen). The enzymes were eluted by a different elution buffer (20 mM phosphate buffer, 150 mM NaCl, 10–400 mM imidazole, 1 mM DTT, pH 7.4). Protein purity and molecular weight were confirmed by SDS-PAGE (10% polyacrylamide) and those fractions containing pure enzyme were pooled, concentrated and buffer exchanged to desalting buffer (50 mM Tris-HCl, 150 mM NaCl, 1 mM DTT, 10% glycerol, pH 8.0) using a centrifugal filter with Amicon Ultra 30 k (Millipore Co.) and stored at −20 °C. SDS-PAGE analysis showed that the target protein had a molecular weight of about 43 kDa, in agreement with the expected CK size (Supplementary Fig. S3).

### Enzyme activity assay and activation energy

For the human uMtCK assays, creatine kinase assay kit (Jiancheng Nanjing Institute of Bio-Engineering, China) was used. According to detecting production of creatine kinase catalysis, human uMtCK enzymatic activity was measured, through phosphomolybdate-based colorimetric method at 660 nm and 30 °C. The *V*_*max*_ of uMtCK was measured by a modified method using the creatine kinase assay kit, uMtCK assays were performed using creatine as substrates at different concentrations (1 mM, 2 mM, 5 mM, 10 mM, 15 mM, 20 mM, and 30 mM) at 25 °C and 30 °C. The value of *V*_*max*_ was calculated with the Michaelis–Menten equation using GraphPad Prism v5.0 (GraphPad Software, USA). The activation energy was calculated based on the methods described in a previous report^34^. Protein concentration was measured using a Protein Quantitative Kit (Bradford) (Applygen Technologies Inc., China) with bovine albumin as a protein standard.

## Additional Information

**How to cite this article**: Li, Q.-J. *et al*. Insights into the Phosphoryl Transfer Mechanism of Human Ubiquitous Mitochondrial Creatine Kinase. *Sci. Rep.* **6**, 38088; doi: 10.1038/srep38088 (2016).

**Publisher's note:** Springer Nature remains neutral with regard to jurisdictional claims in published maps and institutional affiliations.

## Supplementary Material

Supplementary Information

## Figures and Tables

**Table 1:** Summary of the activation energy of uMtCK.

Temperature (K)	Vmax (units/mg)	Activation energy (KJ/mol)
298	432	70.24
303	694	

**Table 2:** Specific Activity for uMtCK, E227A, E227D and E227S.

Protein	Specific Activity (unit/mg)	Protein	Specific Activity (unit/mg)
uMtCK	664 ± 28	E227D	2.6 ± 0.31
E227A	not detected	E227S	3.3 ± 0.28
