# Supplementary material for: Insights into the Phosphoryl Transfer Mechanism of Human Ubiquitous Mitochondrial Creatine Kinase
Source: Sci Rep. 2016 Dec 2;6:38088. doi: 10.1038/srep38088 (PMC5133464; doi:10.1038/srep38088)
Supplement: Supplementary Information [file srep38088-s1.pdf]

Supplementary Information

Insights into the Phosphoryl Transfer Mechanism of Human Ubiquitous Mitochondrial Creatine Kinase

Quanjie Li[a, 1], Shuai Fan [a, 1], Xiaoyu Li[a], Yuanyuan Jin[a], Weiqing He[a], Jinming Zhou[a, *], Shan Cen[a, *], and ZhaoYong Yang[a, *]

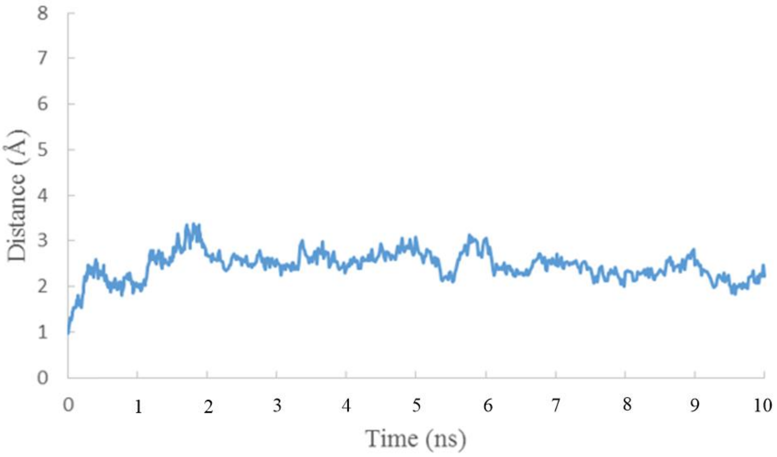

Fig. S1 RMSD values for the complex. Time dependences of root mean square deviation (RMSD) of backbone Cα atoms from the initial structure of uMTCK•ATP-Mg^{2+}•creatine complex.

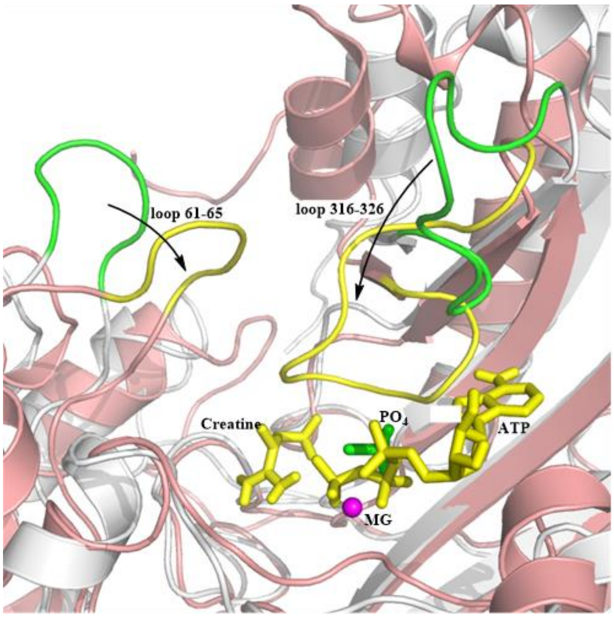

Fig. S2 Alignment of the equilibrated structure (salmon pink) and the crystal structure of human uMtCK (grey). The protein is shown as cartoon while the substrates are depicted as ball-and-stick. Two flexible loops (residue 61-65 and residue 316-326) are highlighted in yellow and green in the equilibrated structure and the crystal structure, respectively. Both loops move into the active site upon substrates binding.

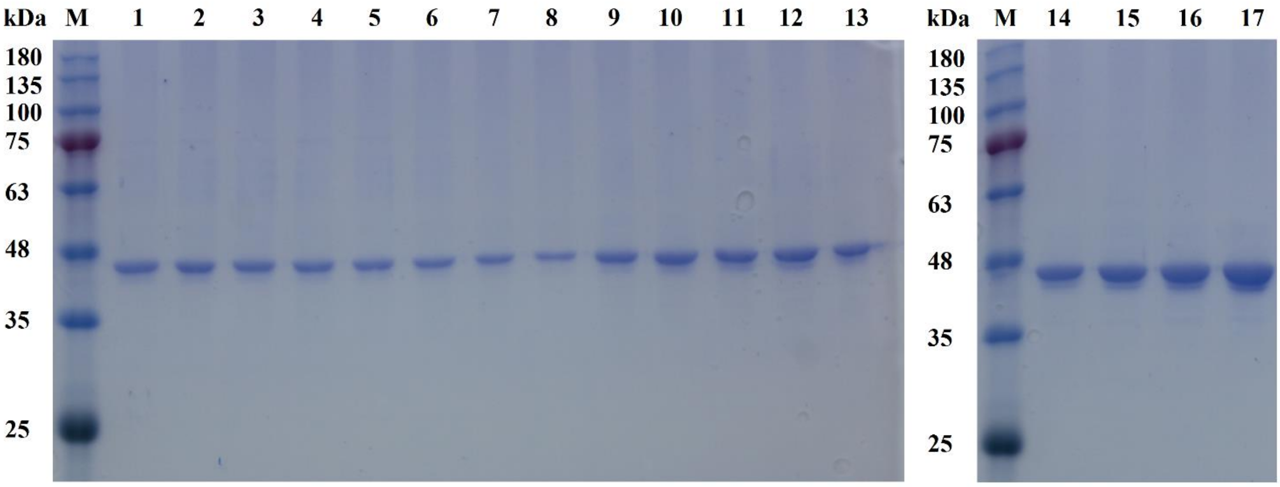

Fig. S3 SDS-PAGE analysis of purified recombinant CK and mutants. The CK and mutants were purified by Ni-NTA agarose gel column equilibrated with the lysis buffer, and their concentrations and purity were determined by Bradford method and SDS-PAGE, respectively. SDS-PAGE analysis showed that the target protein has a molecular weight of about 43 kDa, in agreement with the expected CK size. M: protein molecular weight marker.

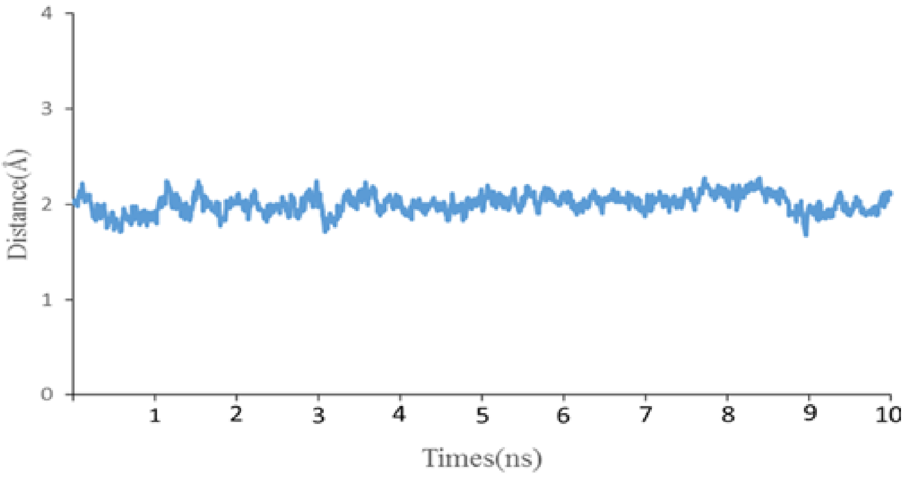

Fig. S4 RMSD values for the complex. Time dependences of root mean square deviation (RMSD) of backbone Cα atoms from the initial structure of E227D•ATP-Mg^{2+}•creatine complex.

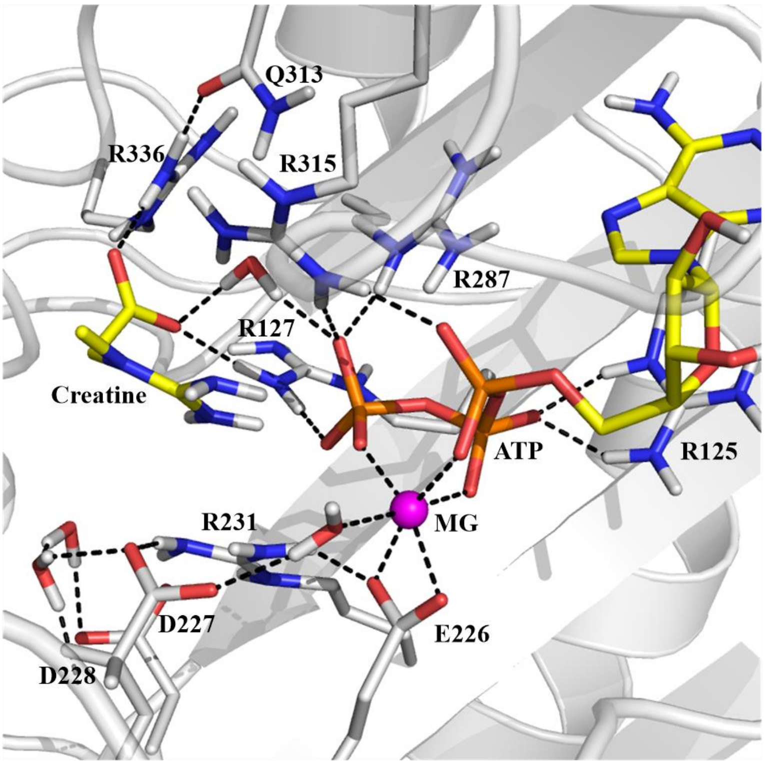

Fig.S5 A 3D Model of the E227D mutant in complex with the co-factor ATP-Mg2+ and creatine. The most striking structural difference between this mutant and the wild-type uMtCK is that D227 located far away from creatine and did not form H-bond with the nucleophilic nitrogen. In particular, the average distances between creatine guanidine group and the carboxylate oxygen atoms of D227 range from 3.9Å to 4.5Å, which is unfavorable for the nucleophilic displacement.

Table S1. Primers for site-directed mutagenesis. Point mutations are underlined.

Site	Name	Sequence (5’ → 3')
H61	ck-H61A-F	GACAACCCGGGTGCTCCGTTTATCA
	ck-H61A-R	TGATAAACGGAGCACCCGGGTTGTC
R125	ck-R125A-F	GTCCTGAGCTCTGCCGTGCGTACCG
	ck-R125A-R	CGGTACGCACGGCAGAGCTCAGGAC
R127	ck-R127A-F	GCTCTCGCGTGGCTACCGGTCGTAG
	ck-R127A-R	CTACGACCGGTAGCCACGCGAGAGC
E226	ck-E226A-F	CTGGGTGAACGCAGAAGATCACACC
	ck-E226A-R	GGTGTGATCTTCTGCGTTCACCCAG

E227	ck-E227A-F	CTGGGTGAACGAAGCAGATCACACC
	ck-E227A-R	GGTGTGATCTGCTTCGTTCACCCAG
	ck-E227S-F	CTGGGTGAACGAATCCGATCACACC
	ck-E227S-R	GGTGTGATCGGATTCGTTCACCCAG
	ck-E227D-F	CTGGGTGAACGAAGATGATCACACC
	ck-E227D-R	GGTGTGATCATCTTCGTTCACCCAG
D228	ck-D228A-F	GTGAACGAAGAAGCTCACACCCGC
	ck-D228A-R	GCGGGTGTGAGCTTCTTCGTTCAC
R231	ck-R231A-F	GAAGATCACACCGCCGTTATCTCC
	ck-R231A-R	GGAGATAACGGCGGTGTGATCTTC
R287	ck-R287A-F	TACGGGTCTGGCTGCAGGTGTGC
	ck-R287A-R	GCACACCTGCAGCCAGACCCGTA
Q313	ck-Q313A-F	CCTGCGCCTGGCCAAGCGTGGTAC
	ck-Q313A-R	GTACCACGCTTGGCCAGGCGCAGG
R315	ck-R315A-F	GCCTGCAAAAGGCAGGTACCGGCGG
	ck-R315A-R	CCGCCGGTACCTGCCTTTTGCAGGC
D321	ck-D321A-F	CCGGCGGTGTCGCCACCGCAGCAAC
	ck-D321A-R	GTTGCTGCGGTGGCGACACCGCCGG
R336	ck-R336A-F	GTAACCTGGACGCACTGGGCAAATC
	ck-R336A-R	GATTTGCCCAGTGCGTCCAGGTTAC

Supplementary Data. Cartesian coordinates for all structures

Re

C	-6.56049900	1.38649900	-1.32270000
N	-5.74720400	0.46975000	-2.10281000
C	-6.07856200	-0.04709000	-3.28280400
N	-7.22713900	0.30300900	-3.90199500
N	-5.24774700	-0.93011500	-3.85481100
H	-6.81028900	2.29183000	-1.89005400
H	-5.97439300	1.69149500	-0.45489900
H	-4.80685200	0.18529300	-1.76884000
H	-7.91055400	0.87142500	-3.42575300
H	-7.55925400	-0.23266900	-4.69053200
H	-4.34372400	-1.07751000	-3.38432600

H	-5.32989300	-1.15288300	-4.83608800
C	-6.34280000	-4.24120000	-0.42220000
C	-4.86190300	-4.55266400	-0.16805400
C	-3.96933000	-4.18706900	-1.36150400
C	-2.46231700	-4.24008700	-1.09467900
N	-1.96090300	-5.60929000	-0.93712100
C	-0.89798900	-5.98616100	-0.19294800
N	-0.39437900	-5.14606600	0.74703200
N	-0.42386300	-7.22489900	-0.31054500
H	-6.49033100	-3.17267700	-0.62582300
H	-4.52041200	-3.99093000	0.71394400
H	-4.73933200	-5.61388400	0.08219900
H	-4.22223900	-4.82247000	-2.22434700
H	-4.18240300	-3.15506700	-1.66587400
H	-1.92041500	-3.73713000	-1.90162100
H	-2.23408600	-3.68748600	-0.18219400
H	-2.20083000	-6.25812400	-1.67738500
H	-0.32508300	-4.15406000	0.48541400
H	0.46898500	-5.48288600	1.16085200
H	-0.43790200	-7.66027300	-1.24742900
H	0.46616800	-7.43206500	0.18779600
C	3.24680000	-4.92670000	-7.72410000
C	2.14996000	-4.09319600	-7.04692100
C	2.51608200	-2.62650300	-6.75594800
C	1.36949700	-1.84605500	-6.09102600
O	0.18754400	-2.15147000	-6.33012900
O	1.70047700	-0.88419800	-5.28272200
H	3.50581900	-4.52286900	-8.71106800
H	1.24724200	-4.10196200	-7.66688500
H	1.87831600	-4.57862100	-6.10150800
H	3.40426200	-2.55705400	-6.11787700
H	2.76946700	-2.11035900	-7.69437600
C	5.15060000	-6.11890000	-4.52670000
C	4.61765700	-5.25322600	-3.37460000
C	4.37972000	-6.03556900	-2.07689600
C	3.78241200	-5.22343800	-0.92146600
O	3.28030000	-4.08643800	-1.16232200
O	3.81317000	-5.76265800	0.23149300
H	6.06029600	-6.65798800	-4.23352500
H	5.32296500	-4.43740200	-3.16996700
H	3.68314900	-4.76705300	-3.67765600
H	3.70964500	-6.89073600	-2.24611100
H	5.31411100	-6.47946400	-1.71078800
C	3.49420000	-9.63220000	-3.92980000

C	2.11359500	-9.01351900	-4.16151800
C	1.39251100	-8.59863500	-2.86943800
O	1.83431900	-9.01167100	-1.78134300
O	0.35845800	-7.83201900	-3.00675500
H	3.41624800	-10.50530400	-3.27655100
H	2.17717600	-8.13255900	-4.81458500
H	1.45779900	-9.72114700	-4.68892200
C	-2.60160000	-7.59480000	-5.07690000
C	-3.17827000	-6.42864200	-5.87780300
C	-2.50002700	-5.08514400	-5.58209400
C	-0.96573600	-5.12376600	-5.72912500
N	-0.23425600	-5.49260800	-4.51457400
C	0.23385600	-4.61916000	-3.60624800
N	0.00406300	-3.30365600	-3.70762000
N	0.88355800	-5.09849400	-2.53656100
H	-2.64045400	-7.40598300	-3.99658900
H	-3.07902500	-6.64620500	-6.95065100
H	-4.25651300	-6.34272700	-5.68514400
H	-2.89267600	-4.32412900	-6.26966100
H	-2.74727400	-4.74430500	-4.56710800
H	-0.68426300	-5.85253000	-6.49714700
H	-0.59825500	-4.16122300	-6.08614400
H	-0.04931200	-6.47907800	-4.28674700
H	-0.23461600	-2.88281300	-4.60535000
H	0.45217700	-2.67190600	-3.04146000
H	0.97752500	-6.11608000	-2.48860500
H	1.53108800	-4.51042800	-2.00691200
C	-2.68520000	-0.72769800	2.56659600
N	-2.61305900	0.33724300	1.57063100
C	-3.10669400	1.55505600	1.75224500
N	-3.54773900	1.93538100	2.97038000
N	-3.27066700	2.38559600	0.69987500
H	-1.89574100	-0.63400600	3.31314100
H	-2.55858300	-1.67702100	2.04376700
H	-2.09643100	0.13169300	0.70598200
H	-3.10088200	1.57559200	3.81806300
H	-4.01209200	2.82891800	3.04714500
H	-2.90878600	2.08934500	-0.21278200
H	-3.16161000	3.37923100	0.85912000
C	-1.16310000	3.03240000	6.27390000
C	-1.03415000	1.59991400	5.80590300
O	-2.00781900	1.01864500	5.28869100
N	0.16928800	1.01625600	5.95157400
H	-1.60571100	3.63557200	5.47483400

H	-0.21737200	3.48374500	6.58597100
H	0.35046300	0.03878400	5.66109200
H	0.90487800	1.49363600	6.45486100
C	-0.31350500	4.46329900	2.20170300
N	-0.16557400	3.09407500	2.70539600
C	0.45054300	2.11208800	1.98812900
N	0.73782000	2.27374200	0.70403700
N	0.78221200	0.95672400	2.58145700
H	-0.78253600	4.45614100	1.21624000
H	0.64618800	4.98674200	2.12142600
H	-0.05202700	3.00553500	3.70536000
H	0.21402600	2.93967400	0.11850100
H	1.06048700	1.40959900	0.23403500
H	0.54914300	0.74274600	3.54025300
H	0.98774600	0.17657700	1.93277300
C	-0.51050400	6.73419700	-2.47690300
C	0.74873400	6.95548200	-1.65717800
O	1.36467100	8.01540400	-1.64369000
H	-1.05643700	7.67841300	-2.54684800
H	-1.15352900	5.95351900	-2.06545400
N	1.16342700	5.86395500	-0.94725800
C	2.45430700	5.87640100	-0.28580100
H	0.65773300	4.99330300	-1.06519700
H	2.49779500	6.67576200	0.46193600
C	-2.15520000	-5.28790000	6.79900000
N	-1.67862100	-4.00060700	6.32842800
C	-2.43170700	-2.91541900	6.23220400
N	-3.69298400	-2.89374600	6.73300500
N	-1.94462800	-1.82672200	5.63091900
H	-1.35389400	-6.01409500	6.65683800
H	-3.02435400	-5.63006700	6.22381500
H	-0.67429800	-3.87873300	6.06284000
H	-4.09799900	-3.73377100	7.11670500
H	-4.33888100	-2.18548300	6.41921800
H	-0.96444000	-1.81585400	5.28545800
H	-2.35703000	-0.90859700	5.75790300
O	0.10401100	-2.58168900	-0.30478600
P	0.50667900	-1.20184300	-0.73697900
O	1.06096700	-1.05528900	-2.16730900
O	1.30034400	-0.36119500	0.28184000
O	-1.02516000	-0.31619500	-0.81129300
P	-1.97376200	-0.06635500	-2.09016500
O	-1.30965500	-0.30153000	-3.42479300
O	-3.33706800	-0.69114900	-1.84559800

O	-2.24229000	1.57029000	-1.88590700
P	-1.08547000	2.73321900	-2.23755700
O	0.15875600	2.02993800	-2.72955100
O	-0.97362400	3.62130200	-1.01676700
O	-1.86363000	3.57853400	-3.36379200
C	-2.54369900	2.91210200	-4.44129600
H	-3.41518100	2.37236700	-4.05842200
H	-1.87665000	2.22298500	-4.96604700
N	3.13902900	-4.27600500	2.38861500
C	3.21019200	-2.94159000	2.26833400
N	3.25917400	-2.40338000	1.04852700
N	3.25244600	-2.13357800	3.36927500
C	3.74016900	-0.75921300	3.26066200
C	2.87523800	-2.61122500	4.69529700
C	1.36830200	-2.52980900	5.04842600
O	0.72537900	-1.46220000	4.75244700
O	0.91345400	-3.52537400	5.64867100
H	3.46049100	-4.70324300	3.24590200
H	3.31156000	-4.84997200	1.51515000
H	2.77530400	-1.51745900	0.88128300
H	3.24260700	-3.05618400	0.23062600
H	2.93129500	-0.02432700	3.28735700
H	4.42132400	-0.56289100	4.09646000
H	4.29721100	-0.64259700	2.33026700
H	3.18951100	-3.64264700	4.85930800
H	3.42036800	-2.00440700	5.42753100
Mg	0.68039000	0.22640200	-3.72391700
O	2.79845500	0.66298800	-3.52778500
H	2.99052900	-0.02949800	-4.19905400
H	2.93508000	0.21699500	-2.67229500
O	0.50780000	1.47998900	-5.46944400
H	1.04886100	0.86453800	-6.00525900
H	1.05044400	2.26873600	-5.30519500
O	2.06019000	-7.88109700	0.74344200
H	2.08381000	-8.46228100	-0.05094300
H	2.77093000	-7.22538500	0.56515800
H	3.27182400	6.04993900	-0.99600300
H	-0.23127400	6.43740800	-3.49507800
H	-2.86903500	3.69937600	-5.12405100
H	-6.72361600	-4.79816000	-1.28704000
H	-6.96387400	-4.50807200	0.44048200
H	-1.55724400	-7.80369000	-5.32551000
H	-3.16412600	-8.51630800	-5.26615900
H	2.91341700	-5.96071200	-7.86514400

H	4.16344900	-4.95151400	-7.12606700
H	5.40185200	-5.50320900	-5.39701200
H	4.41450100	-6.86582200	-4.84410800
H	4.17217500	-8.92462500	-3.44438400
H	3.94798300	-9.94306500	-4.87832400
H	-7.48552200	0.91386200	-0.96844000
H	-3.66005600	-0.71129100	3.06114800
H	-2.40879500	-5.27034400	7.86706000
H	-1.85988300	3.06941600	7.11833600
H	2.61177500	4.91115800	0.20362800
H	-0.95832700	5.01049200	2.89361900

TS1

C	-6.56049900	1.38649900	-1.32270000
N	-5.80824700	0.58012300	-2.26864800
C	-6.20772700	0.25189900	-3.49484500
N	-7.38466300	0.69881000	-3.98801800
N	-5.41438900	-0.52561500	-4.24189500
H	-6.85109300	2.35139700	-1.75662300
H	-5.91104000	1.59105900	-0.47060000
H	-4.86430700	0.22429700	-2.02751400
H	-8.04540600	1.16253900	-3.38361800
H	-7.75676500	0.30572000	-4.83993100
H	-4.49246900	-0.76539700	-3.84488300
H	-5.58964600	-0.65749000	-5.22663200
C	-6.34280000	-4.24120000	-0.42220000
C	-4.84870200	-4.49167800	-0.22841400
C	-4.03845600	-4.09671700	-1.46530300
C	-2.52584000	-4.18179800	-1.28501200
N	-2.06656200	-5.56746500	-1.14758000
C	-1.04650500	-6.01143700	-0.39194400
N	-0.46263700	-5.19105500	0.51783000
N	-0.67147500	-7.29121400	-0.48362800
H	-6.54106600	-3.18203200	-0.63198000
H	-4.48732400	-3.91398800	0.63534200
H	-4.67238300	-5.54671900	0.01646500
H	-4.34795700	-4.71168200	-2.32493500
H	-4.25828700	-3.05720000	-1.73797700
H	-2.02055500	-3.70572200	-2.12811300
H	-2.22919000	-3.63299000	-0.39030400
H	-2.41381500	-6.23159500	-1.82880800
H	-0.34028000	-4.20576900	0.24105200
H	0.39394700	-5.57975300	0.89782500
H	-0.65611000	-7.70329500	-1.42920600
H	0.20144800	-7.53187800	0.02915300

C	3.24680000	-4.92670000	-7.72410000
C	2.19745900	-4.06766200	-7.01116900
C	2.66768000	-2.67916500	-6.54633100
C	1.54488000	-1.87819000	-5.86319200
O	0.35807400	-2.09321700	-6.17856600
O	1.90014400	-1.00770800	-4.97344400
H	3.60187600	-4.44757500	-8.64551600
H	1.33168800	-3.92851600	-7.66676000
H	1.83546600	-4.61996900	-6.13649200
H	3.51567500	-2.75179500	-5.85565400
H	3.02249000	-2.09273500	-7.40761800
C	5.15060000	-6.11890000	-4.52670000
C	4.70735400	-5.18206600	-3.39185000
C	4.35364000	-5.92824200	-2.09416300
C	3.89317500	-5.03711700	-0.94718500
O	4.51725700	-3.91582300	-0.82057200
O	2.98053400	-5.41583600	-0.17452700
H	5.98904700	-6.75362900	-4.21371000
H	5.49853200	-4.45729500	-3.17179700
H	3.83643300	-4.59808700	-3.71771800
H	3.58548700	-6.68963100	-2.25936800
H	5.24446100	-6.46383200	-1.73236500
C	3.49420000	-9.63220000	-3.92980000
C	2.12135600	-9.02282100	-4.22272700
C	1.34802200	-8.60683200	-2.96171100
O	1.71836800	-9.06054300	-1.86275200
O	0.34692100	-7.80589000	-3.13575900
H	3.39318800	-10.50088800	-3.27413500
H	2.20609000	-8.14697800	-4.87979200
H	1.49213800	-9.73987700	-4.76968500
C	-2.60160000	-7.59480000	-5.07690000
C	-3.07013100	-6.50038500	-6.03446500
C	-2.43069500	-5.12961600	-5.78328600
C	-0.89309500	-5.11462400	-5.88541900
N	-0.17050500	-5.48062300	-4.66315400
C	0.14845200	-4.62322300	-3.68408400
N	-0.22792900	-3.33255500	-3.71079100
N	0.83604500	-5.07926700	-2.62688800
H	-2.77375100	-7.30981000	-4.03017400
H	-2.85526400	-6.80712500	-7.06804400
H	-4.16255900	-6.40137800	-5.97071900
H	-2.81822300	-4.41701400	-6.52377700
H	-2.72745600	-4.74148400	-4.79919000
H	-0.56585200	-5.81630000	-6.66050300

H	-0.55096100	-4.12906200	-6.20507900
H	0.04722300	-6.46303100	-4.44482400
H	-0.31975900	-2.82933800	-4.59821600
H	0.09273300	-2.77900700	-2.92182600
H	0.89239300	-6.09432300	-2.50865900
H	1.01163300	-4.44881100	-1.84871900
C	-2.68520000	-0.72770000	2.56660000
N	-2.72699400	0.22493000	1.46408600
C	-3.10746500	1.48698500	1.60490300
N	-3.36645400	1.99381400	2.82673400
N	-3.34254200	2.25201200	0.51088700
H	-1.77653300	-0.61713700	3.16110100
H	-2.69880600	-1.73185900	2.14031600
H	-2.26766500	-0.06720300	0.59086600
H	-2.91689900	1.62624300	3.67086100
H	-3.78982000	2.90815200	2.88818200
H	-3.01450000	1.87705000	-0.38660200
H	-3.11752200	3.23823200	0.58292700
C	-1.16310000	3.03240000	6.27390000
C	-0.97575300	1.60901400	5.80365200
O	-1.87547400	1.03254700	5.16428000
N	0.20791800	1.02662400	6.07986000
H	-1.27862800	3.67758300	5.39676000
H	-0.33522300	3.40954600	6.88041800
H	0.41533500	0.05456800	5.79489900
H	0.85621900	1.48253100	6.70701500
C	-0.31350000	4.46330200	2.20169900
N	0.35514600	3.33340200	2.85063500
C	0.55457800	2.14859700	2.20879300
N	0.14422000	1.95201700	0.97677500
N	1.17271100	1.13977200	2.86559200
H	-1.30362800	4.15174200	1.86260200
H	0.23212500	4.86291600	1.34032500
H	1.02628800	3.55081100	3.57464800
H	-0.16325900	2.68668000	0.31898800
H	0.25409200	0.96796600	0.63860000
H	1.06483800	1.07966200	3.87053800
H	1.17249400	0.24179500	2.36941700
C	-0.51050000	6.73419700	-2.47689800
C	0.72781000	6.95264300	-1.62689300
O	1.32199600	8.02307600	-1.57677400
H	-1.10024200	7.65445100	-2.48503300
H	-1.12166200	5.89577800	-2.13563500
N	1.15278200	5.85126900	-0.92944400

C	2.45430000	5.87640000	-0.28580000
H	0.69216500	4.96312600	-1.10364200
H	2.54685200	6.78171400	0.31917400
C	-2.15520000	-5.28790000	6.79900000
N	-1.73589300	-4.05218000	6.15691700
C	-2.43800900	-2.92540000	6.19746400
N	-3.62571900	-2.88223000	6.85767700
N	-1.96839500	-1.83206300	5.59668600
H	-1.35937900	-6.02129600	6.66395600
H	-3.06688800	-5.69788800	6.34446200
H	-0.82020100	-4.00465200	5.65791800
H	-4.09266300	-3.74362900	7.09860200
H	-4.22295400	-2.07835800	6.73508200
H	-0.96932400	-1.79263100	5.28603900
H	-2.40178700	-0.92371100	5.71370100
O	0.28344500	-2.78158900	-0.78827800
P	0.35411300	-1.27173700	-0.65068100
O	1.12279300	-0.55466400	-1.77012800
O	0.52709300	-0.68565700	0.74619700
O	-1.34065200	-0.76141500	-0.94117500
P	-1.98990800	-0.20288400	-2.29410800
O	-1.12447800	-0.38106000	-3.51639700
O	-3.43634800	-0.66682000	-2.35628000
O	-2.10938800	1.42520600	-1.92438700
P	-1.06824900	2.68900900	-2.27390000
O	0.17358000	2.13844300	-2.92666700
O	-0.94968600	3.51117900	-1.00931800
O	-1.99460300	3.54636000	-3.27930900
C	-2.54369900	2.91210000	-4.44129900
H	-3.32362900	2.19941600	-4.15214200
H	-1.76901500	2.39442500	-5.01570200
N	2.74095300	-4.06702600	2.36726500
C	3.35982000	-2.86195800	2.30320900
N	4.05392100	-2.55370400	1.23386300
N	3.24998600	-2.03412400	3.40428700
C	3.95878100	-0.76282800	3.40581100
C	2.82345700	-2.59418800	4.68941900
C	1.28884400	-2.60763000	4.88109000
O	0.69496000	-1.48281500	4.98965700
O	0.73008300	-3.73043800	4.90846400
H	1.96498300	-4.20793000	3.00206900
H	2.77011700	-4.61886400	1.50241300
H	4.62429500	-1.72142000	1.30711800
H	4.22641700	-3.31840600	0.13094900

H	3.74810300	-0.20692800	2.48786400
H	3.59701200	-0.16156300	4.24139500
H	5.04835300	-0.88376200	3.50926900
H	3.19626300	-3.61613700	4.79758300
H	3.26358800	-1.98678400	5.48570600
Mg	0.82627400	0.26355700	-3.64702500
O	2.93059900	0.72343000	-3.31664200
H	3.18750700	-0.03703200	-3.88005400
H	2.90593700	0.36141200	-2.40963800
O	0.79266900	1.43514000	-5.46617600
H	1.41886700	0.90853700	-5.99328600
H	1.23727700	2.26822400	-5.23910400
O	1.82479100	-7.84227000	0.60168500
H	1.87597700	-8.45765100	-0.16792100
H	2.37756600	-7.08724900	0.30599300
H	3.27734600	5.87725800	-1.01242500
H	-0.20207100	6.53262700	-3.50983900
H	-2.98081000	3.70715600	-5.04896500
H	-6.73638900	-4.82205700	-1.26540000
H	-6.92124200	-4.52000900	0.46623300
H	-1.53707800	-7.82453700	-5.17030100
H	-3.14876000	-8.52834800	-5.25204700
H	2.82572000	-5.90031900	-7.99923600
H	4.12051000	-5.11049500	-7.09068000
H	5.48195600	-5.54473500	-5.39835600
H	4.33729600	-6.77950400	-4.84581800
H	4.14611000	-8.91599400	-3.42055800
H	3.99046700	-9.94572300	-4.85576900
H	-7.45765100	0.86700200	-0.96166600
H	-3.56228900	-0.59633400	3.20456500
H	-2.31454300	-5.15012800	7.87529500
H	-2.08738100	3.10125300	6.85529500
H	2.55837300	4.99527100	0.35498400
H	-0.43209800	5.25703000	2.94207300

Inter

C	-6.56049900	1.38649800	-1.31270000
N	-5.81969800	0.59187500	-2.27709300
C	-6.23314300	0.27800700	-3.50199800
N	-7.41334200	0.73411800	-3.97881600
N	-5.45061200	-0.49544400	-4.26527200
H	-6.85275500	2.35820600	-1.73002100
H	-5.90238700	1.57764000	-0.46411800
H	-4.87424400	0.23005000	-2.04953200
H	-8.06546200	1.19521200	-3.36309300

H	-7.79719700	0.34788700	-4.82867200
H	-4.52561000	-0.74167600	-3.88007200
H	-5.63218000	-0.60857300	-5.25121900
C	-6.35280000	-4.24120000	-0.41220000
C	-4.85344300	-4.48478500	-0.25576800
C	-4.07671500	-4.09331300	-1.51486000
C	-2.55977000	-4.16342800	-1.36645600
N	-2.07850900	-5.54168200	-1.22819700
C	-1.10925500	-5.98755800	-0.41045900
N	-0.59558800	-5.17480800	0.54863600
N	-0.71484500	-7.26196300	-0.49375100
H	-6.56072900	-3.18358700	-0.62052100
H	-4.47248100	-3.90144600	0.59561500
H	-4.66643800	-5.53793300	-0.01078600
H	-4.40062500	-4.71837600	-2.36165200
H	-4.31128500	-3.05748700	-1.79008600
H	-2.07463000	-3.69281900	-2.22459200
H	-2.24712900	-3.60127800	-0.48692400
H	-2.36609700	-6.19594400	-1.94593900
H	-0.42990200	-4.19442700	0.27290800
H	0.21513500	-5.57815600	1.00656500
H	-0.66664100	-7.67159300	-1.43926500
H	0.14895900	-7.48549300	0.04022000
C	3.24680000	-4.92669900	-7.72409900
C	2.20994300	-4.06713000	-6.99442900
C	2.67016800	-2.66078700	-6.57356000
C	1.54700900	-1.86602600	-5.88270200
O	0.36113900	-2.08134800	-6.20235700
O	1.89884500	-1.00445100	-4.98335800
H	3.55696400	-4.46642300	-8.67092600
H	1.32307200	-3.95381200	-7.62579300
H	1.88331400	-4.60607700	-6.09741300
H	3.53617000	-2.70316500	-5.90333500
H	2.99072600	-2.08982900	-7.45823100
C	5.15060000	-6.11890000	-4.52669900
C	4.69631200	-5.17500200	-3.40292800
C	4.41637300	-5.90763000	-2.07815700
C	3.94112200	-5.01315500	-0.94605600
O	4.58548500	-3.88359100	-0.83308600
O	3.02572800	-5.35644200	-0.17763500
H	6.02278600	-6.71005000	-4.22075800
H	5.45972100	-4.41013300	-3.22416200
H	3.78940700	-4.64136600	-3.71574400
H	3.68162700	-6.70868500	-2.20046100

H	5.34429100	-6.38513400	-1.72951600
C	3.49419900	-9.63219900	-3.92980000
C	2.12315600	-9.01149600	-4.20523500
C	1.38650100	-8.55098700	-2.93855200
O	1.80693300	-8.94525700	-1.83415700
O	0.36521400	-7.77672100	-3.11341000
H	3.39819300	-10.48616500	-3.25427300
H	2.20354500	-8.15355000	-4.88639900
H	1.47050400	-9.73180400	-4.71912200
C	-2.60160000	-7.59480100	-5.07690000
C	-3.02719300	-6.51587100	-6.07108600
C	-2.40045600	-5.14047400	-5.81434400
C	-0.86122500	-5.11633800	-5.88526200
N	-0.15833100	-5.46671300	-4.64688700
C	0.15884200	-4.59498900	-3.68019000
N	-0.21702200	-3.30504600	-3.72994500
N	0.84512900	-5.03208200	-2.61269000
H	-2.84215800	-7.30037300	-4.04580100
H	-2.76780400	-6.83792900	-7.08967500
H	-4.12136600	-6.41775000	-6.05599100
H	-2.77537600	-4.43508600	-6.56798700
H	-2.72137100	-4.74726200	-4.83981200
H	-0.51491900	-5.82172600	-6.64863100
H	-0.52140100	-4.13088500	-6.20706600
H	0.06307200	-6.44593000	-4.41744200
H	-0.31431200	-2.81811000	-4.62614000
H	0.11493500	-2.73176900	-2.96159300
H	0.89549400	-6.04487400	-2.47687800
H	0.97316600	-4.39386600	-1.82921100
C	-2.67520000	-0.73769800	2.57659700
N	-2.72838700	0.21084300	1.47045100
C	-3.10378100	1.47443100	1.61132500
N	-3.35308700	1.98474500	2.83337500
N	-3.34438500	2.23847000	0.51721700
H	-1.76486100	-0.61897300	3.16698900
H	-2.68437700	-1.74340800	2.15403200
H	-2.27799600	-0.08350800	0.59414600
H	-2.89877100	1.61883200	3.67624800
H	-3.77345400	2.90051100	2.89427600
H	-3.02191800	1.86014200	-0.38056100
H	-3.10776100	3.22247400	0.58517700
C	-1.16310000	3.03239800	6.28390000
C	-0.96250100	1.61446000	5.80377000
O	-1.85661300	1.03511400	5.15900700

N	0.22516200	1.04035000	6.07789400
H	-1.27917700	3.68418800	5.41162700
H	-0.34128200	3.41138200	6.89751700
H	0.43351000	0.06650500	5.79498200
H	0.86678500	1.49721800	6.71116400
C	-0.30349900	4.46329800	2.22169500
N	0.37913400	3.33213500	2.85339600
C	0.56129900	2.14703200	2.20755800
N	0.12673700	1.95546900	0.98267000
N	1.18668600	1.13481600	2.85128300
H	-1.30836600	4.15831000	1.92087500
H	0.21493100	4.85390500	1.33955500
H	1.05969300	3.54503600	3.56971800
H	-0.17992000	2.69437300	0.33011400
H	0.22286200	0.97119500	0.63930400
H	1.09527500	1.07116500	3.85767900
H	1.18239700	0.23850700	2.35247800
C	-0.51050000	6.73419600	-2.47689800
C	0.72721400	6.95223000	-1.62627800
O	1.32037200	8.02316300	-1.57385700
H	-1.10300600	7.65271800	-2.48050300
H	-1.11934700	5.89245700	-2.13949000
N	1.15390000	5.85003400	-0.93151900
C	2.45429800	5.87640100	-0.28580100
H	0.69239100	4.96216300	-1.10360700
H	2.53972000	6.77307800	0.33321100
C	-2.15520000	-5.28789800	6.79900000
N	-1.74919100	-4.04377800	6.16590900
C	-2.46238200	-2.92461700	6.21455000
N	-3.65132900	-2.89777200	6.87410200
N	-2.00218700	-1.82287500	5.62174400
H	-1.35130500	-6.01153200	6.65880200
H	-3.06243000	-5.70483700	6.34166400
H	-0.83031200	-3.98482300	5.67086700
H	-4.11017700	-3.76579400	7.10677700
H	-4.25715100	-2.09996200	6.75390900
H	-1.00060400	-1.77353100	5.31642900
H	-2.44330700	-0.91996700	5.74682200
O	0.24352700	-2.80837400	-0.74764000
P	0.31395900	-1.29519000	-0.64920400
O	1.09310500	-0.61451800	-1.78475800
O	0.47723200	-0.67712700	0.73442700
O	-1.37524100	-0.79196100	-0.96649900
P	-2.00804800	-0.20630100	-2.31546900

O	-1.13567600	-0.37059500	-3.53510500
O	-3.45670800	-0.65969300	-2.39674300
O	-2.11595000	1.41701200	-1.92104700
P	-1.07363100	2.68106600	-2.26638700
O	0.17678800	2.12634000	-2.90033300
O	-0.96826600	3.51028600	-1.00553200
O	-1.98842500	3.53358600	-3.28635000
C	-2.54369700	2.89210100	-4.44129400
H	-3.33064500	2.19043600	-4.14446000
H	-1.77412400	2.36075600	-5.01004700
N	2.73408800	-3.99399100	2.41008300
C	3.35750800	-2.78309700	2.35336300
N	4.05450700	-2.47373900	1.29383300
N	3.22944300	-1.96906700	3.47276200
C	3.98308400	-0.72488400	3.51653000
C	2.80219200	-2.56110100	4.74328800
C	1.26699400	-2.59568900	4.92175000
O	0.65579600	-1.47943500	5.02781300
O	0.71942900	-3.72440900	4.94258300
H	1.94014400	-4.12907400	3.02296600
H	2.75613900	-4.52406500	1.53696100
H	4.60815900	-1.63088200	1.39122700
H	4.28959200	-3.33469000	0.02696800
H	3.79819200	-0.13361600	2.61510300
H	3.63930900	-0.13535400	4.36862000
H	5.06789000	-0.88545400	3.61960900
H	3.18519500	-3.58082900	4.83763600
H	3.22483800	-1.96360100	5.55659600
Mg	0.82082900	0.25836800	-3.64108100
O	2.92358900	0.69129900	-3.28430200
H	3.18209100	-0.06161400	-3.85684600
H	2.89286800	0.31711500	-2.38243700
O	0.81431000	1.44828500	-5.44662500
H	1.44475400	0.92245700	-5.96964800
H	1.25877500	2.27841300	-5.20884100
O	1.77241900	-7.75080100	0.63776300
H	1.85262800	-8.36089000	-0.13467400
H	2.33311700	-6.99689300	0.35932300
H	3.27729600	5.89518800	-1.01203000
H	-0.20170800	6.53873600	-3.51090600
H	-2.97291400	3.68488300	-5.05749200
H	-6.76551400	-4.82658400	-1.24306100
H	-6.90734700	-4.51910600	0.49165500
H	-1.53093400	-7.81360000	-5.10385800

H	-3.12802200	-8.53621200	-5.27236000
H	2.83358800	-5.91466100	-7.95672500
H	4.14825500	-5.07796200	-7.12157000
H	5.43578000	-5.55187200	-5.41869300
H	4.35843100	-6.81986800	-4.81010900
H	4.16652900	-8.91485800	-3.45003100
H	3.96513600	-9.97147900	-4.85994600
H	-7.45552000	0.86417900	-0.95040400
H	-3.55038200	-0.60921100	3.21772200
H	-2.31625600	-5.16017700	7.87637400
H	-2.09079200	3.08968500	6.86112100
H	2.56491600	4.98655700	0.34157700
H	-0.38917600	5.26236900	2.96078700

TS2

C	-6.56049900	1.38649800	-1.32270000
N	-5.81435400	0.62772900	-2.31095900
C	-6.19537000	0.41282500	-3.56647700
N	-7.35036800	0.93609000	-4.04746200
N	-5.40521600	-0.32129700	-4.35856700
H	-6.84455700	2.37515000	-1.70455400
H	-5.90912200	1.54262800	-0.46169000
H	-4.87765700	0.22105700	-2.08886200
H	-8.02702900	1.32226900	-3.40676700
H	-7.72018000	0.59541100	-4.92287900
H	-4.49107500	-0.60390700	-3.95483000
H	-5.55915600	-0.35701000	-5.35485700
C	-6.34280000	-4.24120000	-0.42220000
C	-4.88087400	-4.53675500	-0.10307500
C	-3.93380900	-3.96523200	-1.16057700
C	-2.45845700	-4.16312600	-0.83539900
N	-2.07014100	-5.57368900	-0.95555600
C	-1.09035300	-6.18735400	-0.27995200
N	-0.47098500	-5.56008800	0.75072400
N	-0.77416400	-7.46052900	-0.56262500
H	-6.52083300	-3.16042400	-0.49130700
H	-4.62362500	-4.10698100	0.87641800
H	-4.73025500	-5.62011800	-0.00753700
H	-4.15736500	-4.40673800	-2.14444600
H	-4.09174100	-2.88697100	-1.27990700
H	-1.83884600	-3.54479900	-1.48319200
H	-2.26082100	-3.84618800	0.19174000
H	-2.44808300	-6.08978700	-1.74056900
H	-0.31426800	-4.55518300	0.64552200
H	0.34544000	-6.07209800	1.07708100

H	-0.72542000	-7.71955100	-1.56158100
H	0.08755200	-7.76150800	-0.09020400
C	3.24680000	-4.92669900	-7.72409900
C	2.20613200	-4.11170600	-6.94764200
C	2.68128200	-2.75353200	-6.40146400
C	1.55333300	-1.99096000	-5.67954900
O	0.38224200	-2.11506300	-6.08822200
O	1.89086500	-1.26198700	-4.66689300
H	3.59208400	-4.38986000	-8.61688200
H	1.33671700	-3.92826500	-7.58694200
H	1.84587700	-4.71524200	-6.10568800
H	3.52745000	-2.86913100	-5.71507300
H	3.03552700	-2.12271900	-7.23096000
C	5.15060000	-6.11890000	-4.52669900
C	4.54035600	-5.15883900	-3.50142100
C	4.31323900	-5.82168300	-2.13261300
C	3.67270000	-4.91493700	-1.10455800
O	4.08756400	-3.67033200	-1.14142200
O	2.84070800	-5.32365600	-0.28176500
H	6.07353000	-6.57334600	-4.14464500
H	5.19217800	-4.28924500	-3.36635200
H	3.58405800	-4.77275100	-3.87564900
H	3.69913500	-6.72439300	-2.20680200
H	5.28164400	-6.13771300	-1.71617800
C	3.49419900	-9.63219900	-3.92980000
C	2.14835300	-8.96633600	-4.22134600
C	1.39372000	-8.50572300	-2.96602100
O	1.80101100	-8.89062700	-1.85208000
O	0.37036700	-7.74046900	-3.16053600
H	3.35952300	-10.50101000	-3.27995200
H	2.26849800	-8.09774000	-4.88286800
H	1.48659900	-9.65537200	-4.76504800
C	-2.60160000	-7.59480100	-5.07690000
C	-3.08103000	-6.54388000	-6.07779000
C	-2.49435800	-5.14512300	-5.85251300
C	-0.95717800	-5.08158400	-5.91413100
N	-0.25922200	-5.42035900	-4.66841600
C	-0.03268000	-4.56247200	-3.66498200
N	-0.50959200	-3.30436000	-3.69058800
N	0.65179100	-4.99018200	-2.59369200
H	-2.80176900	-7.27937400	-4.04371800
H	-2.83184300	-6.87375800	-7.09648600
H	-4.17704100	-6.47906000	-6.04184800
H	-2.88445900	-4.46867200	-6.62470900

H	-2.83008700	-4.73674000	-4.88969200
H	-0.58595800	-5.77921700	-6.67255300
H	-0.63059100	-4.08866800	-6.22900700
H	0.01894500	-6.38857400	-4.46488800
H	-0.55651400	-2.80099400	-4.58297300
H	-0.22105800	-2.69634200	-2.93400700
H	0.77635900	-6.00096500	-2.49346200
H	0.77065900	-4.36228200	-1.79899900
C	-2.68520000	-0.72769800	2.56659700
N	-2.79945200	0.19050600	1.44499400
C	-3.16153200	1.45133700	1.58267100
N	-3.38202000	1.98676700	2.80464400
N	-3.41518800	2.21382200	0.48494000
H	-1.77750500	-0.55134300	3.14811000
H	-2.63645400	-1.73881000	2.16041700
H	-2.38613800	-0.14265200	0.52940300
H	-2.94700400	1.60821100	3.64738300
H	-3.78519600	2.90977100	2.86388400
H	-3.08086600	1.83645700	-0.41064100
H	-3.16330600	3.19424200	0.55226700
C	-1.16310000	3.03239900	6.27390000
C	-0.98094700	1.60205900	5.82791200
O	-1.88110600	1.01774300	5.19936300
N	0.20728400	1.02714200	6.10804200
H	-1.24299700	3.66634400	5.38447700
H	-0.34798400	3.40741900	6.89887600
H	0.42162800	0.05984600	5.82072000
H	0.85387000	1.48922300	6.73218300
C	-0.31349900	4.46329900	2.20169600
N	0.37744100	3.34327300	2.83529800
C	0.50225400	2.12762800	2.23988500
N	0.02034500	1.89455300	1.03733900
N	1.12450800	1.14089800	2.92337900
H	-1.32941300	4.15970000	1.93874700
H	0.18006000	4.83010300	1.29566700
H	1.08329400	3.56333600	3.52408000
H	-0.26979600	2.61806300	0.35056700
H	0.07308500	0.91358900	0.71908600
H	1.05876100	1.14447700	3.93393100
H	1.08319800	0.21423700	2.48407600
C	-0.51050100	6.73419700	-2.47689900
C	0.73234700	6.94408100	-1.63324700
O	1.33245700	8.01187600	-1.58356400
H	-1.12524700	7.63721400	-2.43064800

H	-1.09482500	5.86246900	-2.17385400
N	1.15811300	5.84156500	-0.93959600
C	2.45429900	5.87640000	-0.28580000
H	0.68905700	4.95404100	-1.09734800
H	2.52327300	6.75973900	0.35467500
C	-2.15520000	-5.28789900	6.79900000
N	-1.68999400	-4.05989400	6.17079300
C	-2.39032000	-2.92742400	6.17403600
N	-3.60551400	-2.88177100	6.78185700
N	-1.89245000	-1.83852600	5.59734900
H	-1.36183700	-6.03108500	6.71153700
H	-3.04609100	-5.69142900	6.29992200
H	-0.75844800	-4.02174100	5.71222100
H	-4.08841900	-3.74068000	6.99837100
H	-4.19163900	-2.07356600	6.63547600
H	-0.88950800	-1.80360800	5.29908200
H	-2.33730900	-0.92959200	5.66535000
O	0.40607600	-2.97638300	-0.44683800
P	0.77072900	-1.52754800	-0.27067200
O	1.33194700	-0.75521800	-1.44996500
O	0.50484200	-0.80697000	1.02844700
O	-1.58669800	-0.74711100	-0.77708800
P	-2.04995300	-0.28011300	-2.16740000
O	-1.04940200	-0.52732200	-3.29137400
O	-3.50066300	-0.65870900	-2.49125700
O	-2.17564100	1.42471900	-1.97761200
P	-1.06316200	2.60185400	-2.24260200
O	0.22739100	1.99506700	-2.76187400
O	-0.97014700	3.46244100	-0.99351800
O	-1.82297300	3.50834500	-3.35173400
C	-2.54369700	2.91210100	-4.44129400
H	-3.38524200	2.32264800	-4.06500100
H	-1.89027300	2.27062300	-5.04258700
N	2.27927700	-3.77293500	2.09322400
C	2.82057800	-2.54513300	1.96443600
N	3.06658200	-2.09944000	0.73712300
N	3.07510900	-1.83011200	3.11294600
C	3.92527600	-0.64458000	3.03148500
C	2.85835700	-2.46459200	4.41675400
C	1.36017400	-2.55825200	4.80735900
O	0.74069500	-1.45659200	4.96638200
O	0.86619100	-3.70419000	4.92801800
H	1.87536700	-4.11397200	2.95552100
H	2.24273000	-4.33576000	1.24308400

H	3.55913600	-1.21218000	0.73648500
H	3.63409400	-3.10213600	-0.40378400
H	3.50336700	0.10107600	2.35221500
H	3.97944200	-0.18506200	4.01914100
H	4.94591300	-0.89052300	2.70679800
H	3.30004600	-3.46663400	4.44292900
H	3.36314500	-1.85782500	5.17184900
Mg	0.84956100	0.09482700	-3.44306100
O	2.96715100	0.55254900	-3.16903100
H	3.17024200	-0.22534700	-3.73500400
H	2.99977100	0.20644200	-2.25797400
O	0.80093700	1.33050100	-5.22582700
H	1.53861000	1.14027400	-5.82559300
H	0.97401200	2.19077600	-4.80520500
O	1.81193500	-7.76270500	0.65286500
H	1.88418500	-8.38106700	-0.11222500
H	2.32583600	-6.99658100	0.32331800
H	3.27962600	5.92632200	-1.00769700
H	-0.20994600	6.59734900	-3.52246600
H	-2.90981400	3.73902900	-5.05335500
H	-6.63659400	-4.68354200	-1.38207100
H	-7.01595600	-4.64290500	0.34401600
H	-1.53035800	-7.80137100	-5.14454400
H	-3.12273300	-8.54685300	-5.23099800
H	2.82098700	-5.88035300	-8.05637600
H	4.12818500	-5.15151900	-7.11518600
H	5.40571200	-5.59233200	-5.45147300
H	4.46290400	-6.93197800	-4.78050100
H	4.17545600	-8.94801800	-3.41513200
H	3.97745100	-9.96104600	-4.85727200
H	-7.46255500	0.85625300	-0.98908200
H	-3.55954900	-0.64423100	3.21967500
H	-2.36815700	-5.13928500	7.86427800
H	-2.10326200	3.12224200	6.82579900
H	2.57619800	4.97384600	0.32111900
H	-0.37059200	5.27923000	2.92510400

Pro

C	-6.56049700	1.38649800	-1.32270000
N	-6.00200900	0.65632800	-2.44693400
C	-6.68678100	-0.02933900	-3.34641100
N	-8.03707600	0.05499000	-3.43597800
N	-6.01605600	-0.83789000	-4.18908800
H	-7.05017400	2.31639500	-1.64074800
H	-5.74378300	1.64231400	-0.64621100

H	-4.96183900	0.57180100	-2.58343100
H	-8.55045300	0.60543300	-2.76474400
H	-8.54825400	-0.68308900	-3.89721900
H	-4.98564200	-0.78448600	-4.10675600
H	-6.41660900	-1.03733300	-5.09482800
C	-6.34280000	-4.24120000	-0.42220000
C	-4.97420600	-4.75184600	0.02467400
C	-3.83521100	-4.05760900	-0.72808600
C	-2.43680300	-4.51658100	-0.33065100
N	-2.21057100	-5.90527200	-0.75836500
C	-1.02425200	-6.54394600	-0.65425200
N	0.03169800	-5.92822900	-0.12468000
N	-0.92470700	-7.83208400	-1.01749100
H	-6.42739000	-3.15914300	-0.26285900
H	-4.84956300	-4.58191300	1.10406800
H	-4.92420400	-5.84092200	-0.11428700
H	-3.95836700	-4.20490500	-1.81273800
H	-3.87722600	-2.97244300	-0.58048500
H	-1.69639600	-3.84451800	-0.77131300
H	-2.30885200	-4.47193700	0.75924000
H	-2.83536400	-6.25699000	-1.47144300
H	0.11056900	-4.91538200	-0.21402500
H	0.90311500	-6.48680700	0.02613700
H	-1.75758900	-8.29825500	-1.34872100
H	-0.01432500	-8.16525500	-1.41227800
C	3.24680000	-4.92670000	-7.72410000
C	2.42279800	-3.65864800	-7.45403500
C	2.46388400	-3.21969600	-5.98600300
C	1.44463100	-2.15238000	-5.57257100
O	0.56167300	-1.78998200	-6.39664000
O	1.53824500	-1.71387400	-4.37207500
H	4.30246800	-4.78536000	-7.46313200
H	2.77918500	-2.83921700	-8.09070100
H	1.38161300	-3.82617300	-7.74790100
H	2.29182400	-4.08133900	-5.32796200
H	3.46151900	-2.84790100	-5.71706100
C	5.15050000	-6.11885800	-4.52673100
C	4.36352300	-5.31893800	-3.48537200
C	5.08781500	-5.24903000	-2.12320900
C	4.25423100	-4.51956800	-1.09777400
O	4.22620000	-3.19524500	-1.29176100
O	3.63270700	-5.05548700	-0.19049900
H	6.12818100	-5.66136800	-4.72394600
H	4.19729200	-4.29751400	-3.84213400

H	3.37155300	-5.76648100	-3.34835000
H	5.28716700	-6.25422100	-1.73976800
H	6.04524700	-4.72818200	-2.24311800
C	3.49420000	-9.63220000	-3.92980000
C	2.34229700	-8.77127700	-4.45651700
C	1.52439800	-8.05917400	-3.37016800
O	1.43791200	-8.61642900	-2.23534300
O	0.95089500	-6.97146300	-3.68575500
H	3.12061900	-10.38832100	-3.23402500
H	2.69847900	-8.01791100	-5.16714300
H	1.63675100	-9.40497700	-5.01438600
C	-2.60160000	-7.59480000	-5.07690000
C	-2.77384600	-7.00718600	-6.48318600
C	-2.52479700	-5.49425700	-6.61296100
C	-1.05584400	-5.04289000	-6.53173100
N	-0.44897500	-5.09166800	-5.20164600
C	-0.64684900	-4.19472300	-4.22426300
N	-1.37268000	-3.07743100	-4.44337300
N	-0.17052600	-4.47071100	-3.00253700
H	-3.25533200	-7.07644900	-4.36114200
H	-2.11709900	-7.53992700	-7.18579500
H	-3.79864400	-7.21034900	-6.82136700
H	-2.89515600	-5.16715400	-7.59423200
H	-3.11197900	-4.94617300	-5.86411300
H	-0.43807500	-5.68378300	-7.16943800
H	-0.94981700	-4.02947100	-6.93322400
H	0.13776200	-5.89348100	-4.94287300
H	-1.25528100	-2.65904600	-5.36229600
H	-1.36918400	-2.36232800	-3.70298000
H	0.33600500	-5.35704600	-2.90614200
H	0.05203500	-3.71858300	-2.34522600
C	-2.68520000	-0.72769800	2.56659900
N	-2.98881700	0.20470100	1.49734300
C	-3.29609700	1.46806600	1.69254700
N	-3.39111900	1.99245200	2.93778500
N	-3.60379300	2.25332300	0.62154800
H	-1.81325200	-0.41242600	3.14435600
H	-2.44544500	-1.68348900	2.10231400
H	-2.94892200	-0.16561800	0.49716400
H	-2.97707300	1.53653400	3.74955800
H	-3.61086700	2.97181000	3.03709400
H	-3.19379400	1.93579900	-0.26928600
H	-3.47171600	3.24991700	0.74557900
C	-1.16310000	3.03240000	6.27390000

C	-0.97342900	1.58662700	5.88218700
O	-1.89400700	0.96270700	5.32401200
N	0.23643900	1.04111100	6.12500200
H	-1.22404800	3.63257000	5.35959500
H	-0.35714600	3.42905300	6.89737200
H	0.46347400	0.07381800	5.86574800
H	0.91473300	1.54274200	6.68101400
C	-0.31350300	4.46329900	2.20170100
N	0.33869200	3.28386600	2.78374400
C	0.37172600	2.07398300	2.15875400
N	-0.18430200	1.87868200	0.98901200
N	0.98806900	1.03046800	2.78074600
H	-1.33502800	4.20121500	1.91761000
H	0.19465000	4.85187000	1.31309000
H	1.09599900	3.45395000	3.43152100
H	-0.42232600	2.62929500	0.29561200
H	-0.18245300	0.89456800	0.65130300
H	0.95919800	0.98680600	3.79240500
H	0.82483500	0.13384600	2.29973800
C	-0.51049900	6.73419900	-2.47690000
C	0.72318100	6.94368500	-1.61922500
O	1.30652200	8.02027700	-1.54395500
H	-1.16808100	7.60154500	-2.36859600
H	-1.05328700	5.81640000	-2.24053800
N	1.16087400	5.83492400	-0.94436100
C	2.45430200	5.87640100	-0.28580100
H	0.69930900	4.94426800	-1.11535200
H	2.51535700	6.75848200	0.35732600
C	-2.15520000	-5.28790000	6.79900000
N	-1.60585600	-4.07721300	6.20739900
C	-2.25296000	-2.91366400	6.17190100
N	-3.49764500	-2.80990600	6.70464800
N	-1.67237100	-1.84775900	5.63239400
H	-1.39188500	-6.06493100	6.74485400
H	-3.03649400	-5.64707600	6.25147200
H	-0.65376100	-4.06873500	5.79973600
H	-4.03220700	-3.64227000	6.90122800
H	-4.03012200	-1.96788900	6.54425300
H	-0.67257800	-1.86246200	5.35746600
H	-2.08998900	-0.91945700	5.63997800
O	0.55942400	-3.01911700	-0.52522300
P	0.94359000	-1.56616700	-0.24609200
O	1.45363700	-0.72254900	-1.41469800
O	0.10083000	-0.82678800	0.80000500

O	-3.14671200	-0.77300000	-0.93618400
P	-2.54003700	-0.18287200	-2.20608100
O	-1.22746500	-0.82499800	-2.69376000
O	-3.57311400	0.03682000	-3.32515200
O	-2.15400100	1.39885400	-1.67450200
P	-1.10711000	2.55740500	-2.20228900
O	0.09225000	1.87931300	-2.82695800
O	-0.88256100	3.48291900	-1.01526800
O	-1.96997200	3.44536000	-3.23467500
C	-2.54370100	2.91210100	-4.44129700
H	-3.28554900	2.14598500	-4.20634300
H	-1.77133300	2.46823300	-5.07582900
N	1.71887900	-3.51831900	1.98981000
C	2.48081000	-2.42844300	1.91222500
N	2.55032100	-1.79610100	0.70728100
N	3.15828200	-1.99179200	2.99822600
C	4.02811200	-0.81764000	2.91008500
C	2.97956800	-2.69609500	4.27418000
C	1.52420900	-2.61464900	4.81994000
O	1.03308500	-1.45887800	4.99140100
O	0.95044600	-3.71421000	5.01612000
H	1.47358800	-3.95957900	2.86779000
H	1.18679100	-3.73295500	1.14371300
H	3.07373000	-0.92738900	0.72779200
H	3.58640500	-2.80462800	-0.64558900
H	3.45448000	0.09858700	2.72688400
H	4.55504500	-0.70903800	3.85788300
H	4.78284000	-0.94491100	2.12571900
H	3.26451100	-3.74774800	4.16924600
H	3.65029800	-2.23643600	5.00239600
Mg	0.58364200	-0.07882900	-3.25405200
O	2.69291100	0.48009700	-3.51869300
H	2.82904800	-0.41648900	-3.89577300
H	2.90097300	0.36623900	-2.57410200
O	-0.04321600	0.50615300	-5.22090200
H	0.11351800	-0.28805300	-5.81251600
H	0.53003900	1.22178900	-5.53599600
O	2.29953100	-7.49006400	0.17667500
H	2.21496000	-7.98370600	-0.66848500
H	2.97837000	-6.80419500	0.01718700
H	3.28239100	5.93369000	-1.00438400
H	-0.20155100	6.69164800	-3.52800500
H	-3.00545300	3.75937500	-4.95328100
H	-6.50347800	-4.42823300	-1.49120200

H	-7.15774800	-4.72810900	0.12592500
H	-1.57487500	-7.51507200	-4.70615000
H	-2.87771000	-8.65573300	-5.06808900
H	3.20395500	-5.20740900	-8.78269000
H	2.87028400	-5.77529700	-7.13883000
H	4.60765500	-6.16628700	-5.47456600
H	5.32837000	-7.14652400	-4.19078200
H	4.22973900	-9.02619500	-3.38934600
H	4.01240200	-10.13700700	-4.75255800
H	-7.28241300	0.77332800	-0.76789500
H	-3.54051100	-0.85138400	3.24376800
H	-2.41597100	-5.13879700	7.85353100
H	-2.11226000	3.13954600	6.80569300
H	2.58010300	4.97249200	0.31890900
H	-0.34653700	5.24271000	2.96575600

Reactant (E227D mutant)

C	37.54101900	49.41500500	54.59397100
H	38.06909900	49.27040500	55.54595200
H	36.96748400	48.50894900	54.39260100
N	38.46081900	49.60278700	53.48585500
H	38.46420700	48.94707100	52.67997400
C	39.27968200	50.63974400	53.33804300
N	39.37512600	51.58672300	54.30291400
H	39.04414700	51.38909900	55.23485900
H	40.05762900	52.32518400	54.21843700
N	39.96983300	50.76064700	52.19987500
H	39.84242600	50.01304600	51.49638700
H	40.71637000	51.43187700	52.10602600
C	33.55098300	50.23699600	49.31801200
H	34.45627600	50.81834400	49.53006700
C	33.86941100	49.03813200	48.42466400
H	34.59836200	48.38673800	48.92438100
H	32.96623600	48.43344900	48.26527400
C	34.44680400	49.47893900	47.07746500
H	33.69220400	50.03330200	46.49886700
H	35.26748300	50.18666500	47.25745400
C	35.03706000	48.34603500	46.24206500
H	35.64312000	48.77027100	45.43597800
H	35.70302600	47.74129100	46.85830700
N	34.02577600	47.47871400	45.61301800
H	33.49121500	47.92507600	44.87865100
C	34.03015900	46.12996700	45.55914700
N	34.82655700	45.38489900	46.31189300

H	35.60038800	45.76634600	46.89043800
H	34.62929500	44.37977600	46.35784500
N	33.14106000	45.49693300	44.74047500
H	32.58528500	46.11533700	44.16083500
H	33.56777600	44.74482300	44.15071500
C	43.79900400	46.89600800	43.44101700
H	44.72314100	47.46162000	43.61579900
C	42.90767700	46.86751600	44.68636300
H	42.59998300	47.88859600	44.94811200
H	41.98644300	46.32464600	44.45448700
C	43.55182600	46.20189700	45.90928600
H	43.96837200	45.22465500	45.62995900
H	44.38999200	46.78964200	46.30372200
C	42.57469600	45.92308800	47.04396400
O	41.32574100	46.08125700	46.84957500
O	42.98858300	45.47143600	48.15799300
C	42.05202100	43.70199200	42.18095000
H	41.81136300	44.63681400	42.69694500
C	42.71969200	42.69592700	43.13592100
H	42.91126600	41.75663400	42.60652200
H	43.66838800	43.09471500	43.50681300
C	41.79239300	42.42804700	44.31466300
O	41.98651500	43.09637500	45.37655300
O	40.81886300	41.63398400	44.11584100
C	39.48305100	45.51101700	39.89994000
H	39.80273400	44.46550800	39.89296300
C	37.96614800	45.60694100	39.64293300
H	37.63280100	46.64896800	39.64076200
H	37.74416400	45.17867700	38.65704000
C	37.15473900	44.82998300	40.68478800
O	37.40824100	43.58695200	40.78780000
O	36.34099200	45.47053900	41.41801100
C	37.74199300	50.42298900	44.15499900
H	36.74951600	49.95776700	44.13391900
C	38.81943100	49.49155400	44.73747600
H	39.78075800	50.01859700	44.69984800
H	38.62235600	49.31143200	45.80343200
C	38.98730100	48.13259900	44.02295300
H	38.96531700	48.27107200	42.93351000
H	39.97512800	47.72715800	44.26902400
C	37.91819400	47.09994500	44.42444600
H	37.93550900	46.92546100	45.50696600
H	36.92334400	47.48673200	44.18624500
N	38.00257500	45.81901900	43.71801400

H	37.36504000	45.68300600	42.92485800
C	38.79413200	44.78799800	44.05258600
N	39.61827100	44.85126100	45.09944500
H	39.78175000	45.68949900	45.64600400
H	40.35428200	44.15565100	45.22917200
N	38.71975300	43.63921700	43.35345100
H	38.23841100	43.60293200	42.44856600
H	39.40963400	42.90881000	43.53905500
C	34.21491600	43.63698200	50.94312700
H	34.41857100	42.60539200	51.25920900
H	34.16928100	43.65797400	49.85419500
N	35.26719700	44.55394400	51.33693300
H	35.92287400	44.73066300	50.50892600
C	35.79541000	44.62991000	52.54179800
N	35.29111500	43.92935900	53.59417000
H	34.39580500	43.47482700	53.48827700
H	35.50090400	44.27810300	54.51923800
N	36.86953400	45.42766200	52.76698300
H	37.20713400	45.87313500	51.90961800
H	37.67051300	44.96946000	53.22440300
C	32.64802900	39.14601900	53.26497300
H	31.61289800	39.41426100	53.48945600
H	33.29527700	39.82678600	53.82967800
C	32.90960700	39.33968000	51.78132200
O	32.45119900	40.32754100	51.17695600
N	33.68301400	38.41642800	51.18770600
H	34.02360500	38.53783400	50.22467000
H	34.03710900	37.63337300	51.71746400
C	37.36698500	40.61198400	53.47201500
H	36.95840800	39.79061100	54.06485000
H	36.75655400	41.50575300	53.62682700
N	37.31436500	40.22923700	52.06056200
H	37.43439500	39.25157500	51.83764300
C	37.39579800	41.07990600	51.00398300
N	37.69049500	42.36422700	51.16456600
H	38.32833500	42.73883400	51.89170500
H	37.51558300	43.01302000	50.37899400
N	37.13370500	40.59532500	49.78181100
H	36.50966600	39.80207100	49.61286900
H	37.35588800	41.18550100	48.97514000
C	29.53300300	42.12300900	46.47599400
H	29.56939300	42.62315400	45.50777100
H	28.92872000	42.73345900	47.15638700
N	30.90325500	41.98743400	46.96578200

H	31.67482000	42.27659600	46.37310000
C	31.25024000	41.55229200	48.18997200
N	30.30430200	41.14661000	49.06702000
H	29.38066100	40.89704100	48.74942000
H	30.62733300	40.75572200	49.94679600
N	32.52145800	41.56021100	48.56336200
H	33.25270800	41.86342600	47.88034100
H	32.76330200	41.09012600	49.43754200
O	36.98354000	46.42831000	47.78684600
P	37.66815900	45.46808200	48.74078000
O	38.98466900	44.83554100	48.27250300
O	36.73531700	44.35068100	49.28415300
O	38.01956600	46.33605600	50.16764100
P	39.37219200	47.14752200	50.59885800
O	40.45232800	47.01725000	49.56261100
O	38.95083600	48.52000800	51.08387800
O	39.78507200	46.31843600	51.96295200
P	40.36578800	44.75859800	52.02519600
O	40.84828600	44.35899700	50.64460400
O	39.31005500	43.93444100	52.73699200
O	41.60161200	44.92087600	53.04734800
C	42.79137400	45.59408200	52.60480900
H	42.59240700	46.65972500	52.44644900
H	43.16765600	45.15341900	51.67752500
N	39.49235900	41.24379300	46.44313800
H	40.02940000	41.24911100	45.53884100
H	40.03531600	41.62697200	47.21781200
C	38.22700500	41.68165600	46.39038100
N	37.78033400	42.43579100	47.43569900
H	36.84005700	42.81032200	47.40902700
H	38.43098400	43.16024400	47.78998900
N	37.40471600	41.34079400	45.39147900
C	37.69347400	40.24535400	44.45997600
H	36.96081200	39.44804800	44.62753700
H	37.59891900	40.60733800	43.43362800
H	38.69729600	39.85898600	44.62000300
C	36.11511200	42.01366000	45.23441300
H	36.25596600	43.09741200	45.25106000
H	35.74490700	41.77909900	44.23222000
C	35.06726100	41.58226800	46.27804900
O	35.15338900	40.41970100	46.73131200
O	34.20123700	42.46500000	46.59782400
Mg	40.92787200	45.18071400	48.71698800
O	41.30726200	43.11443300	47.96568700

H	42.11034000	42.86409400	48.45131300
H	41.58206200	43.11718000	47.00132400
O	35.11031000	38.78096400	48.77240400
H	34.94136400	39.45865200	48.05373400
H	35.41646200	38.00416600	48.27869400
O	35.75431100	41.82075500	41.94918100
H	36.37350500	42.48963900	41.52832800
H	35.41764100	41.30486700	41.20078700
O	34.34965900	44.05114000	42.78814700
H	35.02251400	44.60899000	42.32220800
H	34.62864700	43.14051100	42.54772900
H	43.52737800	45.47284700	53.40209600
H	36.84389500	50.25621800	54.69144400
H	33.23634300	43.93820600	51.33771100
H	32.83807400	38.12485500	53.60687300
H	29.05298000	41.14649200	46.33538800
H	32.82889900	50.91172000	48.84133100
H	33.12443500	49.92328600	50.27792700
H	38.38773300	40.82526800	53.81115300
H	37.66226600	51.34406300	44.74337800
H	37.98535900	50.70581100	43.12366100
H	44.08794800	45.88288500	43.13696700
H	43.28071200	47.36293000	42.59562700
H	41.12912800	43.28886000	41.76427100
H	42.71809800	43.94427500	41.34563400
H	40.04244200	46.05060200	39.12813900
H	39.74895100	45.94463700	40.87107800
